# [Br_4_F_21_]^−^ – a unique molecular tetrahedral interhalogen ion containing a μ_4_-bridging fluorine atom surrounded by BrF_5_ molecules†

**DOI:** 10.1039/d3sc06688f

**Published:** 2024-01-20

**Authors:** Martin Möbs, Tim Graubner, Antti J. Karttunen, Florian Kraus

**Affiliations:** a Anorganische Chemie, Fluorchemie, Philipps-Universität Marburg Hans-Meerwein-Str. 4 35032 Marburg Germany f.kraus@uni-marburg.de; b Department of Chemistry and Materials Science, Aalto University 00076 Espoo Finland

## Abstract

The reaction of [NMe_4_][BrF_6_] with an excess of BrF_5_ leads to the compound [NMe_4_][Br_4_F_21_]·BrF_5_. It features molecular [(μ_4_-F)(BrF_5_)_4_]^−^ anions of tetrahedron-like shape containing central μ_4_-bridging F atoms coordinated by four BrF_5_ molecules. It is the most BrF_5_-rich fluoridobromate anion by mass. Quantum-chemical calculations showed that the μ_4_-F–Br bonds within the anion are essentially ionic in nature. The compound is the first example where F atoms bridge μ_4_-like neither to metal nor to hydrogen atoms. It was characterized by Raman spectroscopy and by single-crystal X-ray diffraction. The latter showed surprisingly that its crystal structure is related to the intermetallic half-Heusler compound and structure type MgAgAs.

## Abstract 2

The [(μ_4_-F)(BrF_5_)_4_]^−^ anion in [NMe_4_][Br_4_F_21_]·BrF_5_ is the first example of F atoms bridging μ_4_-like outside ionic solids or [F(HF)_4_]^−^ anions.

## Graphical abstract

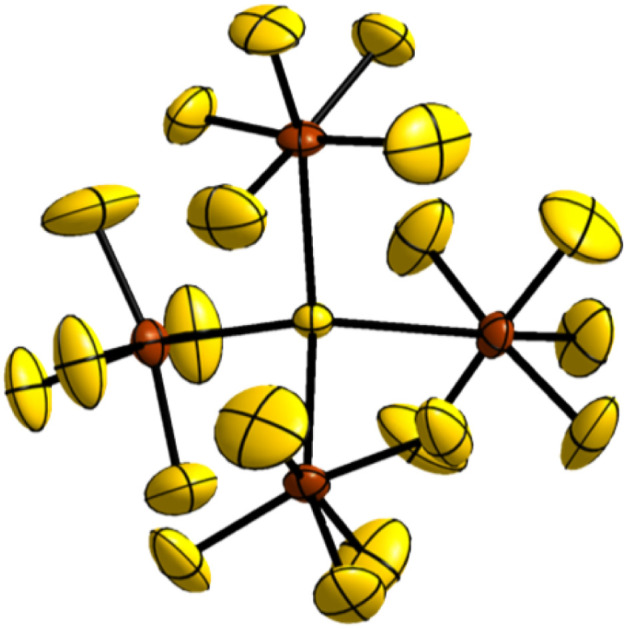

## Introduction

The halogen fluorides form a class of chemically most reactive compounds, some of them even exceeding the reactivity of F_2_ itself. BrF_3_, BrF_5_, and ClF_3_ are hypergolic oxidizers that may react vigorously, sometimes explosively, with many organic and inorganic substrates and materials if conditions are not controlled very carefully.^1,2^ If non-oxidizable starting materials are used, such violent reactions are not to be expected, the respective halogen fluoride is not decomposed and may act as a ligand. Towards Lewis acids, such as BF_3_, AsF_5_ or SbF_5_, the halogen fluorides can act as fluoride ion donors, forming the corresponding fluoridohalonium cations,^3,4^ whereas towards Lewis basic compounds, such as the alkali metal fluorides, many halogen fluorides can act as a fluoride ion acceptors, forming fluoridohalogenate anions.^5,6^ Currently known poly halogen and interhalogen anions are summarized in Table S1.† Currently known poly (inter-) halogen cations are listed in Table S2.† Besides numerous “mononuclear” halogen fluoride ions, only a few examples have been discovered, where halogen fluoride molecules and their derived ions are linked by bridging F atoms forming “oligonuclear” halogen fluoride ions. To the best of our knowledge, only two cations, namely [Br_2_F_8_]^+^ and [Br_3_F_8_]^+^,^7^ and seven anions of this type are currently known. These include [(μ_3_-F)(ClF)_3_]^−^,^8^ [(μ_3_-F)(ClF_3_)_3_]^−^,^9^ [(μ-F)(BrF_3_)_2_]^−^,^10–12^ [(μ_3_-F)(BrF_3_)_3_],^11^ two different isomers of the [Br_4_F_13_]^−^,^13^ [(μ_3_-F)(BrF_5_)_3_]^−^,^14^ and [(μ_3_-F)(IF_5_)_3_]^−^.^15,16^ All those “oligonuclear” anions consist of a central μ- or μ_3_-bridging fluoride ion acting as a linker between two or three halogen fluoride moieties. Depending on the isomer, the [Br_4_F_13_]^−^ anion formally consists of a [(μ_3_-F)(BrF_3_)_3_]^−^ anion with a further BrF_3_ unit attached to one of the terminal F atoms or of a [(μ-F)(BrF_3_)_2_]^−^ anion which is linked to two further BrF_3_ units *via* two different terminal F atoms.^13^

Tetracoordinated F atoms are present in ionic compounds such as CaF_2_. In contrast, only a few complexes with μ_4_-F atoms are known in Y, V, Mo, and Al compounds.^17–21^ Molecular ions containing fluorine atoms that are tetracoordinated by non-metal atoms are generally very scarce, with the tetrahydrogen pentafluoride ion, [(μ_4_-F)(HF)_4_]^−^,^22^ likely being the most prominent example. We recently reported the compound Cs[Br_3_F_16_],^14^ which contained the first fluoride-bridged oligonuclear Br(v) anion, [(μ_3_-F)(BrF_5_)_3_]^−^. It was obtained in the reaction of CsF with an excess of BrF_5_. By variation of the counter ion using KF or RbF instead of CsF as a starting material, we did not succeed in the isolation of other compounds containing fluoride-bridged fluoridobromate(v) anions, as of yet, but obtained the corresponding [BrF_6_]^−^ salts.^14,23^

By introducing the sterically more demanding [NMe_4_]^+^ ion as a counterion, the reaction favors the formation of the bulkier [(μ_4_-F)(BrF_5_)_4_]^−^ ion instead of the smaller [(μ_3_-F)(BrF_5_)_3_]^−^ anion that is formed in the Cs compound. Here we present the first interhalogen compound, [NMe_4_][Br_4_F_21_]·BrF_5_, in which a central fluorine atom is coordinated simultaneously by four halogen fluoride molecules forming a molecular [(μ_4_-F)(BrF_5_)_4_]^−^ anion. The compound was characterized by single-crystal X-ray diffraction and Raman spectroscopy and was investigated by quantum-chemical methods.

## Results and discussion

### Synthesis

The compound tetramethylammonium μ_4_-fluorido-tetrakis(pentafluoridobromate(v))—bromine pentafluoride(1/1), [NMe_4_][Br_4_F_21_]·BrF_5_, was obtained from the reaction of [NMe_4_][BrF_6_] with an excess of BrF_5_. Therefore, [NMe_4_][BrF_6_] was cooled to −196 °C, BrF_5_ was distilled onto the solid, and the reaction mixture was allowed to warm up to room temperature giving a colorless solution. The solution was stored at −36 °C. After several hours, colorless cube-shaped single crystals of [NMe_4_][Br_4_F_21_]·BrF_5_ had formed. The reaction can be described with (Eq. 1).Eq. 1
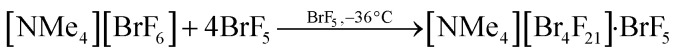


Crystals of the title compound are not stable in BrF_5_ solution at room temperature and decompose to the starting materials. If crystals are warmed slightly above room temperature in the absence of excess BrF_5_, then they decompose under evolution of brown vapors of Br_2_. The left-overs were not characterized further.

When reacting [NMe_4_]F directly with an excess of neat BrF_5_, a very powerful oxidant, the utmost care must be taken. The reaction is hardly controllable and often results in explosions even at temperatures as low as −196 °C (see Fig. S1†). The metathesis reaction between Cs[BrF_6_] and [NMe_4_]F, as reported by Christe and coworkers,^24^ however, offers an elegant and dependable method for obtaining [NMe_4_][BrF_6_], see (Eq. 2).Eq. 2
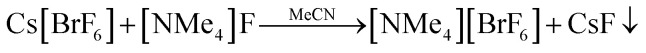


[NMe_4_][BrF_6_] is stable at room temperature, not shock sensitive,^24^ and did not exhibit any violent reaction tendencies when exposed to BrF_5_. Further details on the synthesis are given in the ESI.†

### Crystal structure of [NMe_4_][Br_4_F_21_]·BrF_5_

**Fig. 1:**
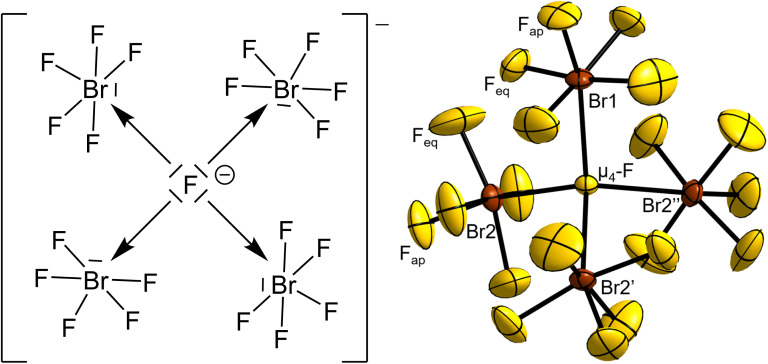
Valence structural formula of the [(μ_4_-F)(BrF_5_)_4_]^−^ anion and its molecular structure in the salt [NMe_4_][Br_4_F_21_]·BrF_5_. Displacement ellipsoids are shown at 50% probability level at 100 K. Symmetry-equivalent, disordered F atoms bound to Br1 are omitted for clarity. Selected bond lengths (Å) and angles (°): μ_4_-F–Br1 2.474(3), μ_4_-F–Br2 2.5447(14), Br1–F_ap_ 1.668(8), Br2–F_ap_ 1.696(3), Br1–F_eq_ 1.685(13) to 1.864(16), Br2–F_eq_ 1.743(5) to 1.783(4), Br1–μ_4_-F–Br2 111.01(7), Br2–μ_4_-F–Br2 107.89(7), μ_4_-F–Br1–F_ap_ 166.6(5), μ_4_-F–Br2–F_ap_ 160.26(18). Symmetry codes: ′*y*, *z*, *x*, ′′*z*, *x*, *y*.

Single-crystal X-ray structure determination showed [NMe_4_][Br_4_F_21_]·BrF_5_ to crystallize in the cubic space group *P*2_1_3 (no. 198, *b*^27^*a*^5^, *cP*192) with the lattice parameter *a* = 13.389(4) Å, *V* = 2400(2) Å^3^, *Z* = 4 at *T* = 100 K. The structure consists of [(μ_4_-F)(BrF_5_)_4_]^−^ fluoridobromate(v) anions (Fig. 1), [NMe_4_]^+^ cations and a BrF_5_ molecule of crystallization per formula unit.

The crystal structure presents a borderline scenario between twinning and/or disorder affecting the cation, a BrF_5_ unit of the anion, and the BrF_5_ molecule of crystallization. These three reside on the threefold rotation axis of the space group and are therefore disordered by symmetry. A corresponding reduction of symmetry by eliminating the threefold rotation axis leads to the orthorhombic space group *P*2_1_2_1_2_1_ and permits a crystallographic description of the structure as a three-component twin. However, the cubic unit cell metric results in too few symmetry-independent reflections to refine the structure properly in the orthorhombic crystal system and to construct a satisfying structural model. Therefore, the choice of the cubic space group *P*2_1_3 with a description of the disorder lead to the clearly superior structure model. Further crystallographic details are given in the ESI.†

The central μ_4_-F atom (4*a*, .3.) of the anion is surrounded tetrahedron-like by BrF_5_ moieties. One BrF_5_ moiety is 2.474(3) Å distant from the μ_4_-F atom with its Br atom residing on a 4*a* position with 3. symmetry, and therefore is affected by disorder over three positions. The remaining three symmetry-equivalent BrF_5_ units reside on the 12*a* position (site symmetry 1), with a μ_4_-F–Br distance of 2.5447(14) Å.

For comparison, the observed distances for the two μ_3_-bridged fluoridohalogenate(v) anions [Br_3_F_16_]^−^^14^ and [I_3_F_16_]^−^^16^ are almost equal with those present here, with *X*–μ_3_-F distances of 2.462(2) Å for *X* = Br and 2.477 to 2.514 Å for *X* = I, respectively. The Br–μ_4_-F–Br angles of the [Br_4_F_21_]^−^ anion are 3 × 111.01(7)° and 3 × 107.89(7)°, showing a small distortion from the ideal tetrahedral angle with 109.5°.

The atomic distances of the Br to the terminally bound F atoms within the BrF_5_ units closely agree with those determined for pure BrF_5_ at 100 K.^23^ The bond lengths between Br2 and the equatorial F atoms, Br2–F_eq_, range from 1.743(5) to 1.783(4) Å and are therefore essentially similar within their tripled standard uncertainties with those in pure BrF_5_ that range from 1.744(3) to 1.779(3) Å. The bond length of the apical Br–F bond, Br2–F_ap_, is slightly longer with 1.696(3) Å compared to 1.686(2) Å for the one in pure BrF_5_, however, there is an overlap of the two bond lengths considering their tripled standard uncertainties. For the disordered BrF_5_ unit, the Br1–F_ap_ bond length is 1.668(8) Å and the Br1–F_eq_ bond lengths range from 1.685(13) to 1.864(16) Å. This broader range and the larger standard uncertainties are caused by the disorder mentioned above. From a chemical point of view, the intramolecular atomic distances and angles of the BrF_5_ units should differ only insignificantly among themselves, which is also confirmed by our quantum-chemical calculations (see below).

The F_ap_–Br–F_eq_ angles of the ordered BrF_5_ units of the anion lie in the range from 83.1(3)° to 84.7(3)°, while for the disordered BrF_5_ unit and BrF_5_ molecule of crystallization they are observed around 83° to 85°. As observed for pure BrF_5_, the Br atoms are located slightly below a virtual plane spanned by the four surrounding F_eq_ atoms by 0.126(7) Å in the case of Br1 and 0.190(3) Å for Br2, respectively. The μ_4_-F–Br–F_ap_ angles are 166.6(5)° for Br1 and 160.26(18)° for Br2 and can be interpreted as an indication for the extent of the stereochemical activity of the free electron pair located on the Br atoms. The more the μ_4_-F–Br–F_ap_ angle deviates from 180°, the stronger the stereochemical influence of the lone pair. In the octahedral [BrF_6_]^−^ anion, no stereochemical effect of the lone pair is observed,^16,23,25–27^ while it is also present in the [(μ_3_-F)(BrF_5_)_3_]^−^ anion, as evidenced in the μ_3_-F–Br–F_ap_ bond angle of 165.0(3)°, also deviating from 180°.^14^ The stereochemical effect of the free electron pair is even more apparent in the analogous [(μ_3_-F)(IF_5_)_3_]^−^ anion, whose μ_3_-F–I–F_ap_ angles are in the range from 141.4° to 149.0°.^16^

**Fig. 2:**
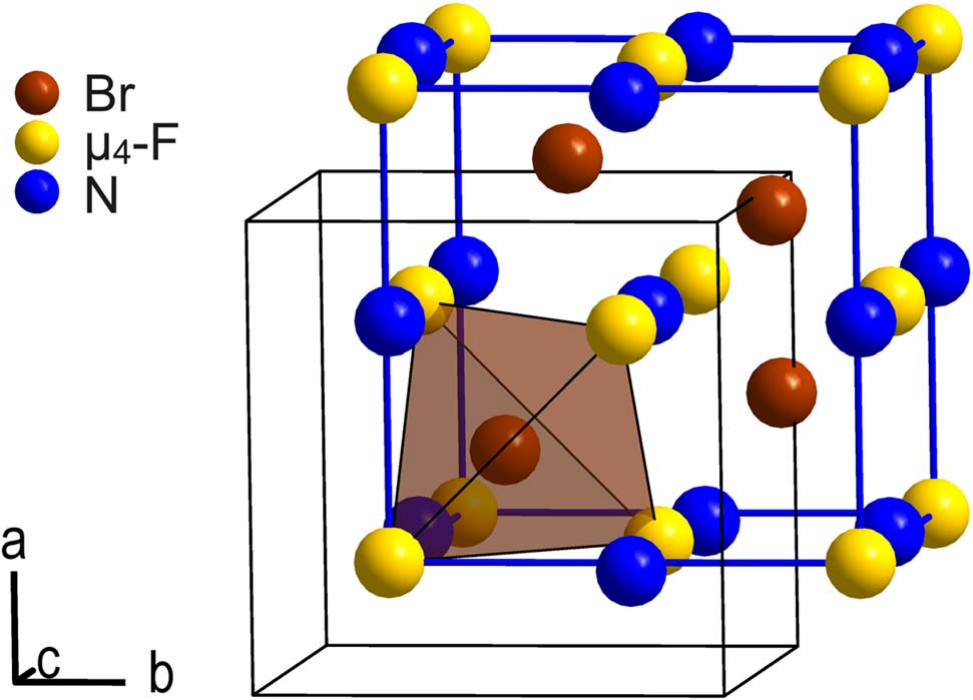
Section of the crystal structure of [NMe_4_][Br_4_F_21_]·BrF_5_ showing the arrangement of the μ_4_-F atoms of the anions (yellow), the N atoms of the [NMe_4_]^+^ cations (blue), and the Br atoms of the BrF_5_ molecules of crystallization (brown) according to the MgAgAs structure type. The pseudo-face centered cubic unit cell is shown in blue. It is shifted by +(1/4, 1/4, 1/4) with respect to the origin of the original unit cell shown in black. Atoms are shown as spheres with arbitrary radii.

Except for Coulomb interaction between the [NMe_4_]^+^ cations and the [Br_4_F_21_]^−^ anions we observe, due to the disorder, no additional intermolecular interactions such as hydrogen bonds in the crystal structure of [NMe_4_][Br_4_F_21_]·BrF_5_. C–H⋯F hydrogen bonds are however likely present as H⋯F distances are overall in the proper range.^28^ Each [Br_4_F_21_]^−^ anion is octahedrally surrounded by [NMe_4_]^+^ cations, and *vice versa*. Thus, cations and anions are arranged in a cubic close packing, respectively, in which all octahedral voids are occupied by counterions. The ionic part of the crystal structure is therefore related to the NaCl structure type. The BrF_5_ molecules of crystallization occupy half of its tetrahedral voids and are arranged tetrahedron-like. So, the overall arrangement of these building blocks corresponds to the intermetallic half-Heusler compound and structure type MgAgAs. Fig. 2 shows the pseudo-face centered cubic unit cell in blue and the arrangement of the N atoms of the cations, the μ_4_-F atoms of the anions, and the Br atoms of the BrF_5_ molecules of crystallization corresponding to the MgAgAs structure type.

### Quantum-chemical calculations on the crystal structure of [NMe_4_][Br_4_F_21_]·BrF_5_

The lattice parameters and atomic positions of the crystal structure were optimized by using hybrid density functional methods (DFT-PBE0/TZVP level of theory, see ESI† for the computational details). Since the structure model in space group *P*2_1_3 derived from the X-ray structure determination includes disordered atoms, the structure was optimized in the orthorhombic subgroup *P*2_1_2_1_2_1_ so that a description of the structure without disorder was possible. A harmonic frequency calculation for the optimized structure confirmed it as a true local minimum on the potential energy surface. The optimized lattice parameters deviate less than 2.3% from the lattice parameter determined by X-ray diffraction and bond lengths and angles are in accordance with the experiment. The calculated four μ_4_-F–Br distances of the [Br_4_F_21_]^−^ anion are in the range of 2.456 to 2.577 Å compared to the experimentally determined distances of 2.474(3) and 2.5446(14) Å. The calculated Br–F_ap_ bond lengths range from 1.704 to 1.708 Å, while the calculated Br–F_eq_ bond lengths are in the range from 1.777 to 1.798 Å. Compared to the experimentally determined bond lengths, the agreement is significantly better for the BrF_5_ units that are not affected by disorder, as expected. For these, the Br–F_ap_ distance was determined to be 1.696(3) Å and the Br–F_eq_ distances are 1.742(5) to 1.783(4) Å. However, the experimentally determined Br–F distances for the disordered parts, which are 1.668(8) Å for the Br–F_ap_ bond and 1.685(13)–1.864(16) Å for the Br–F_eq_ bonds, are of lower precision and therefore spread around the calculated values. A comparison of other calculated and experimentally determined atomic distances and angles is available in Tables S6 and S7.†

### Vibrational spectroscopy

**Fig. 3:**
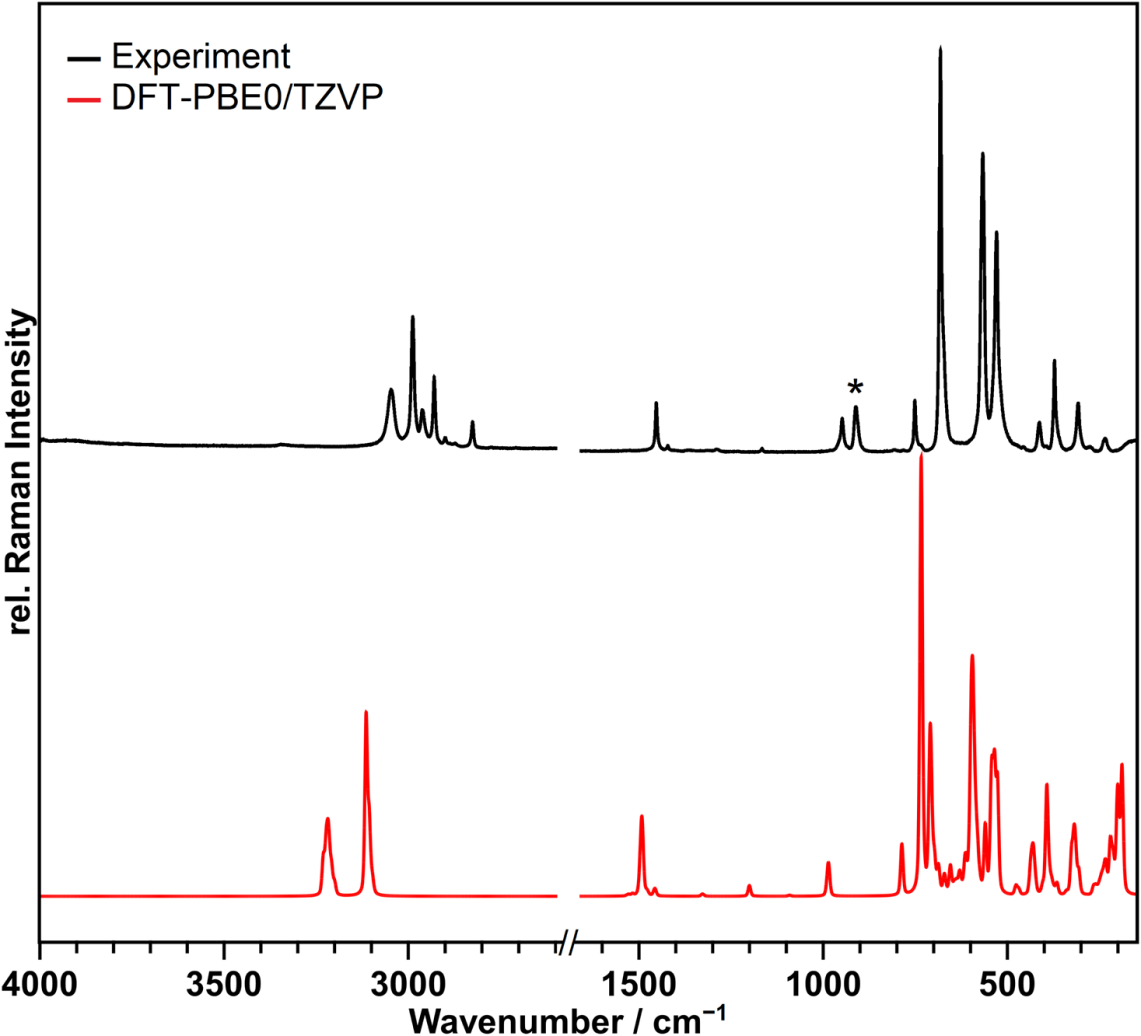
Observed Raman spectrum of [NMe_4_][Br_4_F_21_]·BrF_5_ suspended in perfluorinated oil at 213 K in black and the calculated spectrum at the DFT-PBE0/TZVP level of theory in red. No bands were observed or calculated in the range from 1600–2700 cm^−1^, so it was omitted in the Figure. The band marked with an asterisk originates form BrO_2_F, which is a hydrolysis product formed during the Raman measurement.^29^ The calculated spectrum in red shows many bands not observed in the recorded one. This is because of the lower resolution of the recorded spectrum.

A Raman spectrum of the compound was recorded at 213 K and is shown in Fig. 3 in black in comparison to the calculated spectrum at the DFT-PBE0/TZVP level of theory for the solid-state structure in red.

The bands above 700 cm^−1^ belong to the [NMe_4_]^+^ cation, and those below 700 cm^−1^ to the anion and the BrF_5_ molecule of crystallization, however, the latter two could not be clearly separated. The bands from 529 to 682 cm^−1^ are due to the Br–F stretching vibrations, those from 235 to 413 cm^−1^ are assigned to wagging, scissoring and umbrella modes. Table S8† contains the observed and calculated band positions and their detailed assignments.

With the exception of two points the recorded spectrum agrees with the calculated one in which the frequencies are slightly overestimated as anharmonic effects are not considered.^30^ Additional bands not reproduced by the frequency calculations are observed in the range of 2800–2970 cm^−1^. However, these are assignable to second-order bands of the [NMe_4_]^+^ ion.^31,32^ The band marked with an asterisk at 911 cm^−1^, does not originate from the title compound and also increased in intensity during the measurement. It can be assigned to the symmetric ν(Br–O) stretching vibration of BrO_2_F, which is obtained as a hydrolysis product of the title compound.^29^ The other bands expected for BrO_2_F are significantly weaker in intensity and overlap with the bands of the title compound, so that they cannot be observed. Contact of the sample with the atmosphere causing hydrolysis could not be completely excluded during the transfer of the cold sample into the spectrometer. Additional details of the Raman setup and sample preparation are given in the ESI.†

### Intrinsic bonding orbital analysis

Quantum-chemical calculations on the isolated [(μ_4_-F)(BrF_5_)_4_]^−^ anion and its hypothetical homologues [(μ_4_-F)(ClF_5_)_4_]^−^ and [(μ_4_-F)(IF_5_)_4_]^−^ were conducted at the DFT-PBE0/def2-TZVP level of theory with a consecutive analysis of the bonding situation using intrinsic bonding orbitals (IBOs).^33^ The structures of the molecular anions [(μ_4_-F)(*X*F_5_)_4_]^−^, with *X* = Cl–I, were all optimized for the gas phase at 0 K in point group *S*_4_ and were confirmed to be true local minima on the potential energy surfaces by means of harmonic frequency calculations. The calculated μ_4_-F–Br bond length in the [(μ_4_-F)(BrF_5_)_4_]^−^ anion is 2.576 Å and agrees with the experimentally determined ones of 3 × 2.5447(14) and 2.474(3) Å. The IBO analysis showed that none of the Br atoms contribute to the μ_4_-F–Br bond with more than 1%, which indicates a purely ionic bonding situation, and suggests the description of the μ_4_-F atom as a F^−^ anion. For comparison: In a gas-phase NaF molecule, in which the bond should be highly ionic, the contribution of the F atom is 96%. In the H_2_ molecule, which has a purely covalent bond, the contribution of each H atom is 50%. The partial charge of the μ_4_-F atom is −0.77 *e*, which is significantly more negative than that of the F atoms in a BrF_5_ unit with partial charges between −0.41 and −0.51 *e*. The μ_4_-F atom is even more ionic than the μ_3_-F atom in the [(μ_3_-F)(BrF_5_)_3_]^−^ anion with −0.73 *e*.^14^ The partial charges of the μ_4_-F atoms in the hypothetical anions [(μ_4_-F)(ClF_5_)_4_]^−^ and [(μ_4_-F)(IF_5_)_4_]^−^, calculated to be −0.76 and −0.77 *e*, respectively, are similar to the one observed for [(μ_4_-F)(BrF_5_)_4_]^−^. The partial charges of the Br atoms of the [(μ_4_-F)(BrF_5_)_4_]^−^ anion and the previously reported [(μ_3_-F)(BrF_5_)_3_]^−^ anion are almost the same with +2.38 *e* and +2.37 *e*, respectively.^14^

**Fig. 4:**
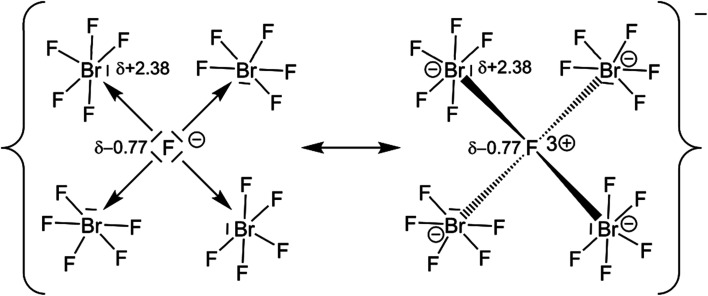
Selected resonance valence structural formulae of the [(μ_4_-F)(BrF_5_)_4_]^−^ anion. Numbers next to the atoms indicate their calculated partial charges. The result of our IBO analysis shows that the one on the left with an essentially ionic Br–F bond is to be preferred.

The high negative partial charge of the μ_4_-F atom as well as the high positive partial charge of the Br atoms of the [(μ_4_-F)(BrF_5_)_4_]^−^ anion strengthen the suggestion of describing the μ_4_-F atom as an F^−^ anion and the μ_4_F–Br bonds as essentially ionic (see Fig. 4).

## Conclusions

The compound [NMe_4_][Br_4_F_21_]·BrF_5_ was obtained by reacting [NMe_4_][BrF_6_] with an excess of BrF_5_. Surprisingly, its crystal structure is closely related to the intermetallic half-Heusler compound and structure type MgAgAs, as the cations, anions and BrF_5_ molecules of crystallization are ordered as in MgAgAs. In the [(μ_4_-F)(BrF_5_)_4_]^−^ anion, the μ_4_-F atom is surrounded by four BrF_5_ molecules in a tetrahedron-like shape. It is the first example of a F atom μ_4_-bridging to neither metal nor H atoms. IBO analyses of the anion showed that the covalence of the μ_4_-F–Br interaction is negligibly small and therefore the bond is best described as ionic. The recorded Raman spectrum of [NMe_4_][Br_4_F_21_]·BrF_5_ agrees with the quantum-chemically calculated one for the solid state. The tetrahedral [(μ_4_-F)(BrF_5_)_4_]^−^ and the propeller-shaped [(μ_3_-F)(BrF_5_)_3_]^−^ anions are so far the only known polynuclear anions of BrF_5_. We have currently no experimental evidence for the existence of the putative dinuclear [(μ-F)(BrF_5_)_2_]^−^ anion.

## Data availability

Upon request from the authors.

## Author contributions

Martin Möbs: planning and conducting the experiments, main data acquisition and interpretation, quantum-chemical calculations and interpretation of the results, manuscript preparation. Tim Graubner: quantum-chemical calculations and interpretation of the results, manuscript preparation. Antti J. Karttunen: quantum-chemical calculations, CRYSTAL basis set development, manuscript preparation. Florian Kraus: project supervision, data interpretation, manuscript preparation.

## Conflicts of interest

The authors declare no conflicts of interest.

## Supplementary Material

SC-015-D3SC06688F-s001

SC-015-D3SC06688F-s002
