# Supplementary material for: [Br4F21]− – a unique molecular tetrahedral interhalogen ion containing a μ4-bridging fluorine atom surrounded by BrF5 molecules
Source: Chem Sci. 2024 Jan 20;15(9):3273–8. doi: 10.1039/d3sc06688f (PMC10901516; doi:10.1039/d3sc06688f)
Supplement: SC-015-D3SC06688F-s001 [file SC-015-D3SC06688F-s001.pdf]

Electronic Supplementary Material (ESI) for Chemical Science.
This journal is © The Royal Society of Chemistry 2024

Supporting Information

Martin Möbs,[a] Tim Graubner,[a] Antti J. Karttunen[b] and Florian Kraus[a]*

[a] M. Sc. M. Möbs, M. Sc. T. Graubner, Prof. Dr. F. Kraus, Anorganische Chemie, Fluorchemie, Philipps-Universität Marburg, Hans-Meerwein-Str. 4, 35032 Marburg, Germany, E-mail: f.kraus@uni-marburg.de

[b] Prof. Dr. A. J. Karttunen, Department of Chemistry and Materials Science, Aalto University, 00076 Espoo, Finland

Table of contents

Table of contents 1

Collection of currently known homo- and hetero polyhalide anions and polyhalogen cations, "mononuclear" and "oligonuclear" species 2

Experimental 3

Single crystal X-ray structure determination 4

Raman Spectroscopy 9

Band assignment for the Raman spectrum of $[NMe_4][Br_4F_{21}]\cdot BrF_5$ 10

Quantum-chemical calculations on the isolated anions $[F(XF_5)_4]^-$ (X = Cl – I) 11

XYZ coordinates for the optimized structures of $[F(XF_5)_4]^-$ anions (X = Cl – I) 11

Solid-state quantum-chemical calculations on $[NMe_4][Br_4F_{21}]\cdot BrF_5$ 13

References 15

Collection of currently known homo- and hetero polyhalide anions and polyhalogen cations, “mononuclear” and “oligonuclear” species

Table S1. Currently known polyhalide anions.

	Homopolyhalide anions	Heteropolyhalide anions	
		“mononuclear”	“oligonuclear”
F	$[F_2]^-$,[1–3] $[F_3]^-$,[4–9] $[F_5]^-$ [9,10]		
Cl	$[Cl_2]^-$[1–3,11] $[Cl_3]^-$,[12–21] $[Cl_5]^-$,[22,23]<br>$[Cl_7]^-$,[24] $[Cl_9]^-$,[19] $[Cl_{11}]^-$,[25] $[Cl_{13}]^-$,[25]<br>$[Cl_8]^{2-}$ [26–28], $[Cl_{12}]^{2-}$,[25]	$[ClF_2]^-$,[5,29–32] $[Cl_2F]^-$,[5]<br>$[ClF_4]^-$,[33–36]<br>$[ClF_6]^-$,[37] $[ClBr_6]^-$,[12]<br>$[ClI_4]^-$ (unsure),[12,38] $[Cl(I_2)_4]^-$,[39–41]<br>$[Cl_2I_{14}]^{2-}$,[42]	$[F(ClF)_3]^-$,[24] $[F(ClF_3)_3]^-$[43]
Br	$[Br_3]^-$,[12,44–48] $[Br_5]^-$,[44,49,46,50,47]<br>$[Br_7]^-$,[42,46,51] $[Br_9]^-$,[46,52–55]<br>$[Br_{11}]^-$,[56,57]<br>$[Br_4]^{2-}$,[58–62] $[Br_6]^{2-}$,[63]<br>$[Br_8]^{2-}$,[64,65,48,42,47] $[Br_{10}]^{2-}$,[66,67]<br>$[Br_{20}]^{2-}$,[42,68] $[Br_{24}]^{2-}$[69]	$[BrF_2]^-$,[32,70,71] $[BrF_4]^-$,[72–80]<br>$[BrF_6]^-$[81–85] $[Br_2Cl]^-$,[86] $[Br(I_2)_2]^-$<br>(unsure),[87] $[BrCl_2]^-$,[12,88–92]	$[F(BrF_3)_2]^-$,[93,80,94] $[F(BrF_3)_3]^-$,[94]<br>$[Br_4F_{13}]^-$,[95] $[F(BrF_5)_3]$, $[Cl(BrCl)_6]^-$,[96]<br>$[Cl(BrCl)_5]^-$,[96] $[Cl(BrCl)_3]^-$,[96]
I	many [97] (I_n^-)<br>many (I_n^{2-}, I_n^{3-}, I_n^{4-}) [98]	$[IF_2]^-$,[32,35,99,100] $[IF_4]^-$,[35,101,102]<br>$[IF_6]^-$,[81,103–105] $[IF_8]^-$,[106–108] $[IF_5]^{2-}$,[109]<br>$[ICl_2]^-$,[41,110–115] $[I_2Cl]^-$,[86,110,116–118]<br>$[ICl_4]^-$,[119–121] $[BrICl]^-$,[122–127] $[I_2Cl_2Br]^-$<br>,[128] $[I_2Br_2Cl_4]^{2-}$, $[I_3BrCl_4]^{2-}$, $[I_4Br_2Cl_2]^{2-}$,<br>$[I_2Br_4Cl_2]^{2-}$, $[I_3Br_3Cl_2]^{2-}$,[129] $[IBr^4]^-$,[47]<br>$[IBr_2]^-$,[130–134] $[I_2Br]^-$,[118,130,135–137]<br>$[I_2Br_6]^{2-}$,[47] $[I_2Br_2]^{2-}$,[118] $[I_2Cl_2]^{2-}$,[118,138]<br>$[I_4Cl_4]^{2-}$,[38,139] $[I_3Cl_5]^{2-}$,[38] $[I_4Cl_8]^{2-}$,[139]<br>$[I_6Cl_6]^{2-}$,[139] $[I_5Br_7]^{2-}$,[134] $[I_2Br_6]^{2-}$,<br>$[I_3Br_5]^{2-}$, $[I_4Br_4]^{2-}$,[140]	$[F(IF_5)_3]^-$,[82,141] $[Cl(ICl)_2]^-$,[121,128,139,142–144]<br>$[Cl(ICl)_3]^-$,[145,139,146] $[I_2Cl_7]^-$ (unsure),[120]<br>$[I_3Cl_{10}]^-$ (unsure),[120] $[Br(IBr)_2]^-$,[131,147]<br>$[Br(IBr)_3]^-$,[148,149] $[I_4Br_5]^-$,[147]

A recent review on polyhalogeno anions is available in the literature.[146]

Table S2. Currently known polyhalogen cations.

	Homopolyhalogen cations	Heteropolyhalogen cations	
		“mononuclear”	“oligonuclear”
F			
Cl	$[Cl_2]^+$, $[Cl_3]^+$,[150–153] $[Cl_4]^+$[154]	$[ClF_2]^+$,[155–158] $[Cl_2F]^+$,[152,159,160]<br>$[ClF_4]^+$,[161,162] $[ClF_6]^+$,[163,164]	
Br	$[Br_2]^+$, $[Br_3]^+$,[165–167] $[Br_5]^+$,[167,168]	$[BrF_2]^+$,[169,170] $[BrF_4]^+$,[171,172] $[BrF_6]^+$,[173]	$[Br_2F_5]^+$,[174] $[Br_3F_8]^+$[174]
I	$[I_2]^+$, $[I_3]^+$,[166,175,176] $[I_5]^+$,[176] $[I_7]^+$<br>(unsure),[177] $[I_4]^{2+}$,[178,179] $[I_{15}]^{3+}$ [180]	$[IF_2]^+$ [181], $[ICl_2]^+$,[182,183] $[IBrCl]^+$,[184]<br>$[IBr_2]^+$,[185] $[I_2Cl]^+$ (unsure),[184,186]<br>$[I_2Br]^+$,[187] $[IF_4]^+$,[171] $[IF_6]^+$,[188]	$[I_4Cl_3]^+$ (unsure),[186] $[I_5Cl_4]^+$ (unsure),[186]<br>$[I_3Cl_2]^+$,[186]

Experimental

All operations were performed on a stainless steel (316L) Schlenk line, which was passivated with fluorine and ClF_3 at various temperatures and pressures before use. Reaction vessels were made out of fluoropolymer (perfluoroalkoxy alkanes, PFA or perfluorinated ethylene propylene copolymer, FEP) and sealed with either a PFA needle valve (Swagelok). The vessels were baked out in vacuum (~10^{-3} mbar) at circa 393 K for several times and passivated with diluted F_2 (F_2/Ar 20:80, V/V, Solvay). All solid starting materials were stored and handled in an Ar-filled (Ar 5.0, Nippon Gases) glove box (MBraun). BrF_5 and $[NMe_4][BrF_6]$ and $[NMe_4]F$ were prepared according to literature.[84,189,190]

Caution! F_2, BrF_5 and the fluoridobromates(V) are highly toxic and very strong oxidizers and therefore must be handled with proper protective equipment and with appropriate emergency treatment procedures available in the event of contact. The utmost precautions must be taken when disposing of these materials and their derivatives.

Synthesis of $[NMe_4][Br_4F_{21}]$: 30.0 mg (0.112 mmol, 1.00 eq) of $[NMe_4][BrF_6]$ was placed in a PFA vessel inside the glove box and an excess of BrF_5 (0.30 mL, 0.74 g, 4.2 mmol, 38 eq) was distilled onto the solid cooled to –196 °C. The reaction mixture was allowed to warm up to room temperature resulting in a colorless solution. Cooling the solution to –36 °C resulted in the formation of colorless cube-shaped crystals of the title compound.

Reaction of $[NMe_4]F$ with neat BrF_5: 55.0 mg (0.590 mmol, 1.00 eq) of NMe_4F was placed in a PFA vessel inside the glove box, sealed with a PFA needle valve, and connected to a Schlenk line. The solid was cooled to –196 °C and BrF_5 vapor was allowed to enter the evacuated vessel via the Schlenk line. Immediately thereafter, an explosion occurred with a sharp bang (see Figure S 1).

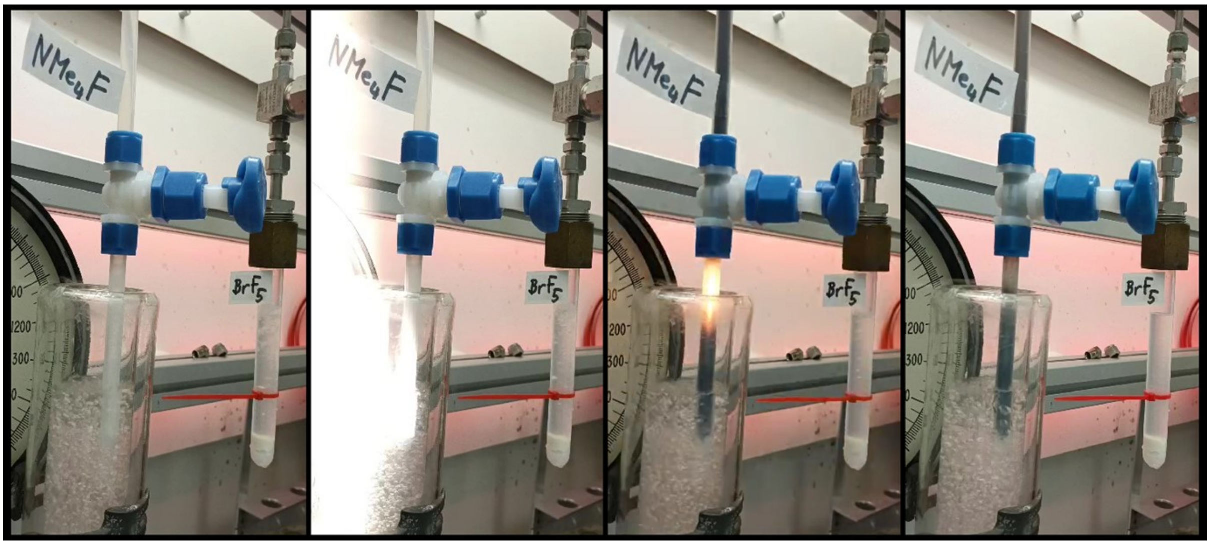

Figure S1. Four consecutive frames of a video of the reaction of $[NMe_4]F$ with BrF_5. The right vessel contains $[NMe_4]F$ and is cooled in a Weinhold vessel filled with liquid nitrogen. On the right side is the vessel containing BrF_5, which is connected to the left vessel via the Schlenk line. As soon as BrF_5 starts to condense in the left reaction vessel, it starts reacting. A bright flash (second frame) followed by an orange-yellow flame shooting upwards through the vessel (third frame) can be seen. After the reaction, the vessel is still intact and only covered by a dense layer of soot (fourth frame) that can be wiped off again.

Single crystal X-ray structure determination

Single-crystal X-ray diffraction of $[NMe_4][Br_4F_{21}]\cdot BrF_5$: Crystals of $[NMe_4][Br_4F_{21}]\cdot BrF_5$ were selected in the absence of air under dried, cooled perfluoropolyether (Galden LS/230, Solvay, stored over molecular sieves 3 Å) and mounted on a MiTeGen loop. Intensity data of a suitable crystal were recorded with a D8 Quest diffractometer (Bruker). The diffractometer was operated with monochromatized Mo-K_α radiation (0.71073 Å) using multi-layered optics and equipped with a PHOTON III C14 detector. Evaluation, integration and reduction of the diffraction data was carried out with the APEX3 software suite.[191] The diffraction data was corrected for absorption utilizing the numerical absorption correction method of SADABS within the APEX3 software suite. The structure was solved with dual-space methods (SHELXT) in space group $P2_13$ (No. 198),[192] and refined against F^2 using the SHELXL program within the ShelXle suite as a merohedral twin by introducing the following twin instruction: TWIN 0 –1 0 –1 0 0 0 0 1.[193,194]

Because of the BrF_5 moieties as well as the $[NMe_4]^+$ ion located at 4*a* positions with .3. symmetry, those are affected by disorder. Alternatively, a structural model in the orthorhombic subgroup $P2_12_12_1$ (No. 19), without threefold rotation axis, was considered. For this purpose, the detwinned data were first extracted from the fcf-file created with the LIST 8 option of SHELXL using the HKLF5Tools program,[195] which allowed to introduce the additional threefold twin instruction (TWIN 0 0 1 1 0 0 0 1 0 3) for the following refinement in space group $P2_12_12_1$. However, a stable refinement in space group $P2_12_12_1$ was only possible with

hard constraints such as rigid bonds or fixed atomic positions and fixed batch scale factors, and still resulted in ill-behaved or non-positive atomic displacement parameters, strong correlations between atom coordinates, and therefore worse *R*-values than for the refinement in space group $P2_13$, which therefore is the superior choice. The description of the structure as disordered is also reasonable because the recorded reflection intensities decrease sharply at higher diffraction angles, which would not be expected if the structure were merely twinned by classical pseudo-merohedry.

Representations of the crystal structures were created with the Diamond software.[196] Non-hydrogen atoms were refined with anisotropic displacement parameters. Anisotropic displacement parameters of fluorine atoms affected by disorder were restrained to approximate isotropic behavior by introducing appropriate ISOR instructions when necessary. H atom positions of the disordered methyl groups were calculated and refined using a riding model (AFIX) with isotropic displacement parameters 1.5 times U_{eq} of the associated carbon atom. The highest residual electron density after the final refinement was 0.45 e·Å^{-3}, 0.89 Å distant from atom F2E. CCDC 2311055 contains the supplementary crystallographic data for this paper. These data are provided free of charge by The Cambridge Crystallographic Data Centre.

Table S3. Selected crystallographic data and details of the structure determination of $[NMe_4][Br_4F_{21}]\cdot BrF_5$.

Compound	$[NMe_4][Br_4F_{21}]\cdot BrF_5$
Molar mass / g·mol^{-1}	967.70
Space group (No.)	$P2_13$ (198)
a / Å	13.389(4)
V / Å^3	2400(2)
Z	4
Pearson symbol	*cP*192
$\rho_{calc.}$ / g·cm^{-3}	2.678
μ / mm^{-1}	8.57
Color	colorless
Crystal morphology	block
Crystal size / mm^3	0.47 × 0.43 × 0.33
T / K	100
λ / Å	0.71073
No. of reflections measured	61002
θ range / °	2.151 – 33.745
Range of Miller indices	$-20 \leq h \leq 20$, $-20 \leq k$ 20, $-20 \leq l \leq 20$
Absorption correction	numerical
T_{max}, T_{min}	0.205, 0.099
R_{int}, R_{σ}	0.0504, 0.0207
Completeness of the data set	1
No. of unique reflections	3221
No. of parameters	179
No. of restraints	54*
S (all data)	1.035
$R(F)$ ($I \geq 2\sigma(I)$, all data)	0.0248, 0.0273
$wR(F^2)$ ($I \geq 2\sigma(I)$, all data)	0.0645, 0.0655
BASF	0.466(2)
$\Delta\rho_{max}$, $\Delta\rho_{min}$ / e·Å^{-3}	0.45, –0.52

*ISOR restraints on U_{ij} parameters of disordered F atoms.

Table S4. Wyckoff position, site symmetry, fractional atomic coordinates, and isotropic or equivalent isotropic displacement parameters (Å^2).

	Wyck.	*site*	*x*	*y*	*z*	*Uiso*/Ueq*	*Occ.* (<1)
Br1	4*a*	.3.	0.14698 (2)	*x*	*x*	0.02843 (14)	
Br2	12*b*	1	0.36683 (3)	0.36404 (3)	0.14815 (3)	0.02639 (9)	
Br3	4*a*	.3.	0.46870 (3)	*x*	*x*	0.04010 (19)	
F1	4*a*	.3.	0.25366 (14)	*x*	*x*	0.0219 (6)	
F1A	12*b*	1	0.063 (5)	0.068 (7)	0.1003 (16)	0.060 (6)	0.3333
F1B	12*b*	1	0.0745 (18)	0.2406 (15)	0.0737 (17)	0.095 (5)	0.3333
F1C	12*b*	1	0.2078 (15)	0.1291 (14)	0.0320 (11)	0.064 (3)	0.3333
F1D	12*b*	1	0.2104 (15)	0.0518 (9)	0.1995 (13)	0.060 (3)	0.3333
F1E	12*b*	1	0.0635 (17)	0.1600 (18)	0.2447 (14)	0.084 (5)	0.3333
F2A	12*b*	1	0.4714 (3)	0.4090 (3)	0.0926 (4)	0.0652 (11)	
F2B	12*b*	1	0.4249 (4)	0.2464 (3)	0.1311 (4)	0.0701 (15)	
F2C	12*b*	1	0.3197 (6)	0.3578 (6)	0.0270 (4)	0.104 (3)	
F2D	12*b*	1	0.3326 (4)	0.4912 (3)	0.1526 (4)	0.0709 (15)	
F2E	12*b*	1	0.4412 (4)	0.3724 (7)	0.2583 (4)	0.089 (2)	
F3A	12*b*	1	0.540 (5)	0.558 (3)	0.524 (3)	0.059 (6)	0.3333
F3B	12*b*	1	0.5094 (14)	0.3800 (14)	0.5603 (13)	0.068 (4)	0.3333
F3C	12*b*	1	0.5874 (12)	0.441 (2)	0.4087 (16)	0.083 (5)	0.3333
F3D	12*b*	1	0.440 (2)	0.5670 (15)	0.3977 (15)	0.073 (5)	0.3333
F3E	12*b*	1	0.3747 (13)	0.4948 (19)	0.5498 (12)	0.067 (4)	0.3333
N1	4*a*	.3.	0.7352 (3)	*x*	*x*	0.0259 (9)	
C1	4*a*	.3.	0.6707 (3)	*x*	*x*	0.0356 (16)	
H1A	12*b*	1	0.606095	0.660379	0.703476	0.053*	0.3333
H1B	12*b*	1	0.703476	0.606095	0.660379	0.053*	0.3333
H1C	12*b*	1	0.660379	0.703476	0.606095	0.053*	0.3333
C2	12*b*	1	0.8380 (8)	0.7201 (12)	0.7011 (12)	0.054 (3)	0.5
H2A	12*b*	1	0.881164	0.771676	0.730023	0.081*	0.5
H2B	12*b*	1	0.840396	0.724519	0.628034	0.081*	0.5
H2C	12*b*	1	0.861346	0.654055	0.722285	0.081*	0.5
C3	12*b*	1	0.8160 (9)	0.7917 (16)	0.6765 (9)	0.062 (4)	0.5
H3A	12*b*	1	0.854520	0.833795	0.722373	0.093*	0.5
H3B	12*b*	1	0.784289	0.833760	0.625653	0.093*	0.5
H3C	12*b*	1	0.860688	0.743697	0.644068	0.093*	0.5

Table S5. Anisotropic displacement parameters (Å^2).

	U^{11}	U^{22}	U^{33}	U^{12}	U^{13}	U^{23}
Br1	0.02843 (14)	0.02843 (14)	0.02843 (14)	−0.00650 (14)	−0.00650 (14)	−0.00650 (14)
Br2	0.02618 (18)	0.02442 (17)	0.02858 (18)	−0.00481 (13)	0.00501 (14)	0.00427 (14)
Br3	0.04010 (19)	0.04010 (19)	0.04010 (19)	−0.00966 (15)	−0.00966 (15)	−0.00966 (15)
F1	0.0219 (6)	0.0219 (6)	0.0219 (6)	−0.0010 (7)	−0.0010 (7)	−0.0010 (7)
F1A	0.049 (12)	0.068 (14)	0.063 (11)	−0.033 (6)	−0.010 (16)	−0.02 (2)
F1B	0.089 (5)	0.083 (9)	0.113 (11)	0.039 (7)	−0.053 (8)	−0.003 (8)
F1C	0.089 (5)	0.066 (7)	0.038 (5)	−0.014 (7)	0.022 (6)	−0.012 (6)
F1D	0.089 (5)	0.025 (4)	0.065 (8)	0.009 (6)	−0.014 (7)	0.004 (5)
F1E	0.089 (5)	0.093 (11)	0.069 (9)	−0.032 (9)	0.032 (7)	−0.021 (8)
F2A	0.052 (2)	0.062 (3)	0.081 (3)	−0.0139 (19)	0.036 (2)	0.013 (2)
F2B	0.064 (3)	0.0406 (19)	0.106 (4)	0.0095 (19)	0.040 (3)	0.005 (2)
F2C	0.125 (6)	0.131 (7)	0.058 (3)	−0.012 (5)	−0.052 (4)	−0.002 (4)
F2D	0.082 (3)	0.0261 (14)	0.104 (4)	0.0013 (17)	0.045 (3)	0.008 (2)
F2E	0.072 (3)	0.145 (6)	0.049 (3)	0.010 (4)	−0.021 (2)	−0.018 (3)
F3A	0.064 (9)	0.052 (10)	0.061 (11)	−0.026 (8)	−0.023 (8)	−0.012 (7)
F3B	0.065 (7)	0.081 (8)	0.059 (7)	−0.014 (7)	−0.019 (6)	0.013 (6)
F3C	0.045 (7)	0.113 (12)	0.091 (10)	0.001 (9)	0.013 (7)	0.012 (9)
F3D	0.087 (10)	0.060 (8)	0.072 (9)	−0.034 (8)	−0.004 (8)	0.014 (7)
F3E	0.072 (8)	0.084 (9)	0.045 (6)	0.014 (8)	0.010 (6)	−0.013 (7)
N1	0.0259 (9)	0.0259 (9)	0.0259 (9)	0.0012 (10)	0.0012 (10)	0.0012 (10)
C1	0.0356 (16)	0.0356 (16)	0.0356 (16)	−0.0034 (14)	−0.0034 (14)	−0.0034 (14)
C2	0.026 (4)	0.071 (9)	0.064 (8)	−0.015 (5)	0.004 (5)	−0.015 (7)
C3	0.029 (5)	0.120 (14)	0.037 (5)	−0.034 (7)	0.002 (4)	0.010 (6)

Table S6. Selected experimentally observed and calculated equivalent atomic distances of $[NMe_4][Br_4F_{21}]\cdot BrF_5$ in Å (DFT-PBE0/TZVP for solid-state and DFT-PBE0/def2-TZVP for single molecule). Note that the site symmetry of the disordered anion in the crystal structure is C_3, while in the solid-state quantum-chemical calculation the point group is C_1 and S_4 in the molecular calculation.

	d(exp.) / Å	*d*(solid state calc.) / Å	*d*(single molecule calc.) / Å
Br1–F1A	1.668(8)	1.706	1.715
Br1–F1B	1.864(16)	1.791	1.792
Br1–F1C	1.759(13)	1.792	1.794
Br1–F1D	1.685(13)	1.781	1.786
Br1–F1E	1.729(17)	1.782	1.779
Br2–F2A	1.696(3)	1.704, 1.708, 1.706	-
Br2–F2B	1.771(4)	1.784, 1.785, 1.777	-
Br2–F2C	1.742(5)	1.801, 1.787, 1.789	-
Br2–F2D	1.764(4)	1.793, 1.781, 1.785	-
Br2–F2E	1.783(4)	1.798, 1.795, 1.779	-
Br1–F1	2.474(3)	2.462	2.576
Br2–F1	2.5446(14)	2.456, 2.510, 2.577	

Table S7. Selected experimentally observed and calculated angles for the $[Br_4F_{21}]^-$ anion in °. Symmetry operations for generation of equivalent atoms: ' *z, x, y*. Note that the site symmetry of the disordered anion in the crystal structure is C_3, while in the solid-state quantum-chemical calculation the point group is C_1 and S_4 in the molecular calculation. DFT-PBE0/TZVP level of theory for solid-state and DFT-PBE0/def2-TZVP for single molecule.

	∡(exp.) /°	∡(solid state calc.) /°	∡(single molecule calc.) /°
Br1-F1-Br2	111.01(7)	112.06, 108.13, 109.11	110.567
Br2'-F1-Br2	107.89(7)	106.83, 109.75, 110.95	108.926
F1A-Br1-F1	166.6(5)	155.19	155.629
F1A-Br1-F1E	84.8(17)	83.34	83.823
F1A-Br1-F1B	83.(4)	84.34	83.262
F1A-Br1-F1C	84.2(12)	83.85	83.911
F1A-Br1-F1D	91.(4)	83.44	83.351
F1B-Br1-F1	102.5(6)	111.54	110.839
F1C-Br1-F1	108.5(6)	113.74	115.041
F1C-Br1-F1B	82.5(8)	90.66	89.581
F1D-Br1-F1	84.5(5)	79.73	80.778
F1D-Br1-F1C	91.7(8)	88.91	89.259
F1D-Br1-F1E	94.9(8)	88.94	89.466
F1D-Br1-F1B	172.1(8)	167.75	167.733
F1E-Br1-F1C	167.2(9)	167.17	166.610
F1E-Br1-F1	83.0(7)	72.28	77.751
F1E-Br1-F1B	89.8(11)	88.77	88.840
F2A-Br2-F2D	83.5(2)	83.45	-
F2A-Br2-F2E	83.1(3)	84.90, 82.44, 83.23	-
F2A-Br2-F1	160.28(18)	162.14, 154.40, 154.84	-
F2A-Br2-F2C	84.7(3)	84.94, 83.84, 83.13	-
F2A-Br2-F2B	84.1(2)	83.59, 84.16, 83.86	-
F2B-Br2-F1	79.43(14)	78.57, 79.00, 82.58	-
F2B-Br2-F2E	85.3(3)	88.71, 88.32, 88.49	-
F2C-Br2-F2D	89.0(3)	90.36, 90.71, 89.36	-
F2C-Br2-F2B	89.8(4)	88.43, 87.85, 89.36	-
F2C-Br2-F1	105.9(3)	95.36, 114.38, 117.74	-
F2C-Br2-F2E	167.2(3)	169.69, 166.06, 166.33	-
F2D-Br2-F2E	93.3(3)	90.21, 90.10, 89.13	-
F2D-Br2-F1	112.78(14)	114.39, 112.93, 108.56	-
F2D-Br2-F2B	167.6(2)	167.04, 167.34, 167.59	-
F2E-Br2-F1	84.8(3)	93.78, 77.96, 75.34	-

Raman Spectroscopy

The Raman spectra were recorded with a Monovista CRS+ confocal Raman microscope (Spectroscopy & Imaging GmbH) using a 488 nm solid-state laser for excitation and either a 300 grooves/mm (low-resolution mode, FWHM: <5.50 cm^{-1}) or a 1800 grooves/mm (high-resolution mode, FWHM: <0.443 cm^{-1}) grating. Sample preparation of $[NMe_4][Br_4F_{21}]\cdot BrF_5$: Crystals of the compound were selected under extensive exclusion of air under dried, cooled perfluoropolyether (Galden LS/230, Solvay, stored over molecular sieves 3 Å) using an optical microscope. For the transfer to the Raman microscope, the crystals within the oil were cooled with liquid nitrogen and quickly transferred onto the pre-cooled microscopy stage (*BCS196, Linkam*) of the Raman microscope. During the measurement, the sample chamber was flushed with a stream of nitrogen. However, air contact cannot be completely prevented when transferring the sample, which can also be seen from the fact that ice crystals form during the measurement on the object slide carrying the sample. The spectrum was recorded at a temperature of –60 °C in order to avoid the increase of hydrolysis products. After the measurement, the crystals of $[NMe_4][Br_4F_{21}]\cdot BrF_5$ suspended in perfluoropolyether were allowed to slowly warm up on the cooled sample holder and their decomposition was visually observed under the light microscope. At about –30 °C the crystals began to shrink and droplets of a colorless liquid formed on the surfaces of the crystals. As the temperature further increased, the crystals began to dissolve more and more. A Raman spectrum of the remaining crystals recorded at 5 °C indicated that the compound was still present. At about 22 °C, the remaining crystals and the liquid that had formed began to react vigorously, forming a brownish liquid indicative of the formation of bromine. However, since the presence of moisture could not be completely excluded and the hydrolysis was occurring simultaneously, the decomposition temperature of $[NMe_4][Br_4F_{21}]\cdot BrF_5$ could not be reliably determined.

Band assignment for the Raman spectrum of $[NMe_4][Br_4F_{21}]\cdot BrF_5$

Table S8. Band assignment for the calculated Raman spectrum (>150 cm^{-1}) of $[NMe_4][Br_4F_{21}]\cdot BrF_5$. The notation for band assignments is the following: ν – stretching, δ – deformation, s symmetric, as – asymmetric. vw – very weak, w -weak, m – medium, s -strong, vs – very strong, sh – shoulder. The band assignment is derived from visual inspection from the calculated normal modes using the visualization tool CRYSPLOT.[197,198]

ν(observed) / cm^{-1}	ν(calculated) / cm^{-1}	Assignment
235(vw)	233	BrF_5: wagging $\delta(F_{eq}–Br–F_{eq})$
308(w)	306, 318, 325	BrF_5: scissoring mode $\delta(Br–F_{eq})$
372(m)	393	BrF_5: umbrella mode $\delta(Br–F_{eq})$
413(w)	430	BrF_5: wagging $\delta(F_{ap}–Br–F_{eq})$
529(s)	528, 535, 542, 560	BrF_5: various $Br–F_{eq}$ stretching vibrations
567(s)	595, 616	BrF_5: out of phase streching $\nu_s(Br–F_{eq}) + \nu_{as}(Br–F_{eq})$
673(sh)	710	BrF_5: out of phase stretching $\nu_s(Br–F_{ap})$
682(vs)	734	BrF_5: in phase stretching $\nu_s(Br–F_{ap})$
751(w)	786	NMe_4: $\nu_s(N–C)$
948(w)	985	NMe_4: $\nu_{as}(N–C)$
1166(vw)	1200	NMe_4: $\delta_{as}(CH_3)$
1289(vw)	1328	NMe_4: $\delta_{as}(CH_3)$ combined with $\delta_{as}(N–C)$
1422(vw)	1457	NMe_4: $\delta_s(CH_3)$
1453(w)	1493	NMe_4: $\delta_{as}(CH_3)$
2826(w)<br>2872(vw)<br>2900(vw)<br>2929(m)<br>2962(w)	–	NMe_4: 2nd order vibrations[199]
2988(m)	3197, 3106, 3114	NMe_4: $\nu_s(CH_3)$
3047(w)	3200, 3208, 3218, 3223, 3231,	NMe_4: $\nu_{as}(CH_3)$

Quantum-chemical calculations on the isolated anions $[F(XF_5)_4]^-$ (X = Cl – I)

Molecular calculations were carried out with the TURBOMOLE7.7[200,201] program suite using the PBE0 hybrid density functional method (DFT-PBE0).[202,203] We used the def2-TZVP triple-ζ valence basis set for fluorine, chlorine, bromine, and iodine atoms.[204] For the iodine atom, scalar relativistic effects were taken into account using a 28-electron effective core potential (ECP).[205] Multipole-accelerated resolution-of-the-identity approximation (MA-RIJ) was used to speed up the DFT calculations[206–208] and an *m4* integration grid was used for the numerical integration of the exchange correlation part. The "COnductor-like Screening MOdel" (COSMO) was applied to all structures to compensate the negative charge.[209] The structures of the complex anions were fully optimized within the constraints of their respective molecular point group symmetry. Numerical harmonic frequency calculations were performed to check if the optimized structures were true local minima on the potential energy surfaces. Intrinsic bond orbital (IBO) analyses were performed with the Turbomole module proper.[210]

XYZ coordinates for the optimized structures of $[F(XF_5)_4]^-$ anions (X = Cl – I)

$[Cl_4F_{21}]^-$ in S_4

F	0.0000000	0.0000000	0.0000000
Cl	1.4691575	-1.4586392	-1.4873217
Cl	-1.4691575	1.4586392	-1.4873217
Cl	-1.4586392	-1.4691575	1.4873217
Cl	1.4586392	1.4691575	1.4873217
F	2.1831973	-2.4970614	-2.4752505
F	0.0343204	-2.1245352	-2.0375236
F	1.4787249	-0.4034414	-2.8004424
F	3.0407930	-0.9641672	-1.0980627
F	1.5856654	-2.6758668	-0.3314059
F	-2.1831973	2.4970614	-2.4752505
F	-0.0343204	2.1245352	-2.0375236
F	-1.4787249	0.4034414	-2.8004424
F	-3.0407930	0.9641672	-1.0980627
F	-1.5856654	2.6758668	-0.3314059
F	-2.4970614	-2.1831973	2.4752505
F	-2.6758668	-1.5856654	0.3314059
F	-0.9641672	-3.0407930	1.0980627
F	-0.4034414	-1.4787249	2.8004424
F	-2.1245352	-0.0343204	2.0375236
F	2.4970614	2.1831973	2.4752505
F	2.1245352	0.0343204	2.0375236
F	2.6758668	1.5856654	0.3314059
F	0.9641672	3.0407930	1.0980627
F	0.4034414	1.4787249	2.8004424

$[Br_4F_{21}]^-$ in S_4

F	0.0000000	0.0000000	0.0000000
Br	1.6742868	-1.2960479	-1.4669919
Br	-1.6742868	1.2960479	-1.4669919
Br	-1.2960479	-1.6742868	1.4669919
Br	1.2960479	1.6742868	1.4669919
F	2.2306125	-2.6181823	-2.4066603
F	0.1229204	-1.6517722	-2.2607476
F	2.1555726	-0.3254904	-2.8945717
F	3.3728472	-1.2647918	-0.8895561
F	1.3226982	-2.5565745	-0.2521715
F	-2.2306125	2.6181823	-2.4066603
F	-0.1229204	1.6517722	-2.2607476
F	-2.1555726	0.3254904	-2.8945717

F	-3.3728472	1.2647918	-0.8895561
F	-1.3226982	2.5565745	-0.2521715
F	-2.6181823	-2.2306125	2.4066603
F	-2.5565745	-1.3226982	0.2521715
F	-1.2647918	-3.3728472	0.8895561
F	-0.3254904	-2.1555726	2.8945717
F	-1.6517722	-0.1229204	2.2607476
F	2.6181823	2.2306125	2.4066603
F	1.6517722	0.1229204	2.2607476
F	2.5565745	1.3226982	0.2521715
F	1.2647918	3.3728472	0.8895561
F	0.3254904	2.1555726	2.8945717

$[I_4F_{21}]^-$ in S_4

F	0.0000000	0.0000000	0.0000000
I	1.7209019	-1.1989673	-1.5837829
I	-1.7209019	1.1989673	-1.5837829
I	-1.1989673	-1.7209019	1.5837829
I	1.1989673	1.7209019	1.5837829
F	2.0783513	-2.8187833	-2.3796741
F	-0.0289470	-1.6234400	-2.2042688
F	2.1220805	-0.5891590	-3.3410817
F	3.5832479	-1.3028446	-1.2040848
F	1.4244682	-2.3550036	-0.0964825
F	-2.0783513	2.8187833	-2.3796741
F	0.0289470	1.6234400	-2.2042688
F	-2.1220805	0.5891590	-3.3410817
F	-3.5832479	1.3028446	-1.2040848
F	-1.4244682	2.3550036	-0.0964825
F	-2.8187833	-2.0783513	2.3796741
F	-2.3550036	-1.4244682	0.0964825
F	-1.3028446	-3.5832479	1.2040848
F	-0.5891590	-2.1220805	3.3410817
F	-1.6234400	0.0289470	2.2042688
F	2.8187833	2.0783513	2.3796741
F	1.6234400	-0.0289470	2.2042688
F	2.3550036	1.4244682	0.0964825
F	1.3028446	3.5832479	1.2040848
F	0.5891590	2.1220805	3.3410817

Solid-state quantum-chemical calculations on $[NMe_4][Br_4F_{21}]\cdot BrF_5$

Solid-state calculations on $[NMe_4][Br_4F_{21}]\cdot BrF_5$ were performed with the CRYSTAL23[211] program suite using hybrid density functional methods (DFT-PBE0)[202,203] combined with triple-valence basis sets TZVP for all atoms[212–214] (derived from molecular Karlsruhe basis sets[204]). Empirical D3 dispersion correction with zero damping[215,216] was applied for the geometry optimization and frequency calculation to take into account weak intermolecular interactions. Atomic positions and lattice parameters were fully optimized within the space group symmetry. As a starting point for the optimization the orthorhombic structure model (space group $P2_12_12_1$, No. 19) was used in order to be able to consider the BrF_5 molecules without the issue of crystallographic disordering. The reciprocal space was sampled with a 2×2×2 Monkhorst-Pack-type *k*-point grid.[217] Tight truncation criteria (TOLINTEG 8, 8, 8, 8, 16) were applied for the evaluation of the bielectronic Coulomb and exchange series in all calculations. Default DFT integration grids and optimization convergence thresholds were used in all calculations. The optimized lattice parameters deviated by less than 2.3% from the lattice parameters determined with SCXRD. Harmonic vibrational frequencies and Raman intensities were calculated with the schemes implemented in CRYSTAL.[218–220] The harmonic frequencies confirmed the optimized structure as a true local minimum. Raman intensities were calculated for a polycrystalline powder sample, applying the same conditions as in the experimental set (T = 213.15 K and λ = 488 nm). For the simulation of the Raman spectrum, a pseudo-Voigt band profile (50:50 Lorentzian:Gaussian) with a FWHM of 8 cm^{-1} was used. The band assignment is derived from visual inspection from the calculated normal modes using the visualization tool CRYSPLOT.[198]

In the following section, atomic positions and lattice parameters of the optimized structure are given in CIF format:

```
data_findsym-output
_audit_creation_method FINDSYM

_cell_length_a    13.2028547200
_cell_length_b    13.0867608600
_cell_length_c    13.1904647400
_cell_angle_alpha 90.0000000000
_cell_angle_beta  90.0000000000
_cell_angle_gamma 90.0000000000
_cell_volume      2279.0828245102

_symmetry_space_group_name_H-M "P 21 21 21"
_symmetry_Int_Tables_number 19
_space_group.reference_setting '019:P 2ac 2ab'
_space_group.transform_Pp_abc a,b,c;0,0,0

loop_
_space_group_symop_id
_space_group_symop_operation_xyz
1 x,y,z
2 x+1/2,-y+1/2,-z
3 -x,y+1/2,-z+1/2
4 -x+1/2,-y,z+1/2
```

```
loop_
_atom_site_label
_atom_site_type_symbol
_atom_site_symmetry_multiplicity
_atom_site_Wyckoff_label
_atom_site_fract_x
_atom_site_fract_y
_atom_site_fract_z
_atom_site_occupancy
_atom_site_fract_symmform
C1  C    4 a 0.65818 0.66772 0.66727 1.00000 Dx,Dy,Dz
H1  H    4 a 0.64553 0.70811 0.59628 1.00000 Dx,Dy,Dz
H2  H    4 a 0.58748 0.65921 0.70856 1.00000 Dx,Dy,Dz
H3  H    4 a 0.69120 0.59308 0.65237 1.00000 Dx,Dy,Dz
C2  C    4 a 0.74859 0.67539 0.82832 1.00000 Dx,Dy,Dz
H4  H    4 a 0.78069 0.60058 0.81302 1.00000 Dx,Dy,Dz
H5  H    4 a 0.67697 0.66761 0.86798 1.00000 Dx,Dy,Dz
H6  H    4 a 0.80112 0.72089 0.87295 1.00000 Dx,Dy,Dz
C3  C    4 a 0.68662 0.83144 0.74996 1.00000 Dx,Dy,Dz
H7  H    4 a 0.61489 0.82264 0.78942 1.00000 Dx,Dy,Dz
H8  H    4 a 0.67476 0.86983 0.67780 1.00000 Dx,Dy,Dz
H9  H    4 a 0.73956 0.87471 0.79609 1.00000 Dx,Dy,Dz
C4  C    4 a 0.82779 0.73880 0.67482 1.00000 Dx,Dy,Dz
H10 H    4 a 0.85663 0.66263 0.65926 1.00000 Dx,Dy,Dz
H11 H    4 a 0.88100 0.78096 0.72187 1.00000 Dx,Dy,Dz
H12 H    4 a 0.81460 0.77971 0.60437 1.00000 Dx,Dy,Dz
Br1 Br   4 a 0.36673 0.36377 0.15553 1.00000 Dx,Dy,Dz
Br2 Br   4 a 0.35947 0.14860 0.36945 1.00000 Dx,Dy,Dz
Br3 Br   4 a 0.13042 0.36198 0.36771 1.00000 Dx,Dy,Dz
Br4 Br   4 a 0.45891 0.46880 0.47586 1.00000 Dx,Dy,Dz
Br5 Br   4 a 0.15057 0.14723 0.14174 1.00000 Dx,Dy,Dz
F1  F    4 a 0.25111 0.25276 0.25896 1.00000 Dx,Dy,Dz
F2  F    4 a 0.33513 0.37013 0.02394 1.00000 Dx,Dy,Dz
F3  F    4 a 0.41940 0.24048 0.13176 1.00000 Dx,Dy,Dz
F4  F    4 a 0.42901 0.36273 0.27640 1.00000 Dx,Dy,Dz
F5  F    4 a 0.34070 0.49650 0.16983 1.00000 Dx,Dy,Dz
F6  F    4 a 0.48205 0.40705 0.11554 1.00000 Dx,Dy,Dz
F7  F    4 a 0.49186 0.15481 0.33972 1.00000 Dx,Dy,Dz
F8  F    4 a 0.34740 0.03337 0.29585 1.00000 Dx,Dy,Dz
F9  F    4 a 0.23720 0.12357 0.42123 1.00000 Dx,Dy,Dz
F10 F    4 a 0.37508 0.24841 0.46187 1.00000 Dx,Dy,Dz
F11 F    4 a 0.39941 0.06934 0.46389 1.00000 Dx,Dy,Dz
F12 F    4 a 0.24745 0.42941 0.36983 1.00000 Dx,Dy,Dz
F13 F    4 a 0.14888 0.32940 0.49785 1.00000 Dx,Dy,Dz
F14 F    4 a 0.00099 0.32253 0.37616 1.00000 Dx,Dy,Dz
F15 F    4 a 0.10365 0.42028 0.24907 1.00000 Dx,Dy,Dz
F16 F    4 a 0.08404 0.47337 0.41636 1.00000 Dx,Dy,Dz
F17 F    4 a 0.50980 0.57187 0.53745 1.00000 Dx,Dy,Dz
F18 F    4 a 0.57929 0.46250 0.41716 1.00000 Dx,Dy,Dz
F19 F    4 a 0.50831 0.39947 0.58009 1.00000 Dx,Dy,Dz
F20 F    4 a 0.35042 0.49727 0.54889 1.00000 Dx,Dy,Dz
F21 F    4 a 0.42187 0.56149 0.38584 1.00000 Dx,Dy,Dz
F22 F    4 a 0.05332 0.12270 0.06016 1.00000 Dx,Dy,Dz
F23 F    4 a 0.05241 0.19788 0.21954 1.00000 Dx,Dy,Dz
F24 F    4 a 0.12185 0.02245 0.18988 1.00000 Dx,Dy,Dz
F25 F    4 a 0.22795 0.09262 0.04425 1.00000 Dx,Dy,Dz
F26 F    4 a 0.15538 0.26603 0.07602 1.00000 Dx,Dy,Dz
N1  N    4 a 0.73011 0.72835 0.73009 1.00000 Dx,Dy,Dz

# end of cif
```

References

[1] T. G. Castner, W. Känzig, *J. Phys. Chem. Solids* **1957**, *3*, 178–195.
[2] W. F. Howard, L. Andrews, *J. Am. Chem. Soc.* **1973**, *95*, 3045–3046.
[3] W. F. Howard, L. Andrews, *Inorg. Chem.* **1975**, *14*, 409–413.
[4] B. S. Ault, L. Andrews, *J. Am. Chem. Soc.* **1976**, *98*, 1591–1593.
[5] B. S. Ault, Lester. Andrews, *Inorg. Chem.* **1977**, *16*, 2024–2028.
[6] A. A. Tuinman, A. A. Gakh, R. J. Hinde, R. N. Compton, *J. Am. Chem. Soc.* **1999**, *121*, 8397–8398.
[7] F. A. Redeker, H. Beckers, S. Riedel, *RSC Adv.* **2015**, *5*, 106568–106573.
[8] S. Riedel, T. Köchner, X. Wang, L. Andrews, *Inorg. Chem.* **2010**, *49*, 7156–7164.
[9] T. Vent-Schmidt, F. Brosi, J. Metzger, T. Schlöder, X. Wang, L. Andrews, C. Müller, H. Beckers, S. Riedel, *Angew. Chem., Int. Ed.* **2015**, *54*, 8279–8283.
[10] F. Brosi, T. Vent-Schmidt, S. Kieninger, T. Schlöder, H. Beckers, S. Riedel, *Chem. Eur. J.* **2015**, *21*, 16455–16462.
[11] M. Hass, D. L. Griscom, *J. Chem. Phys.* **1969**, *51*, 5185–5186.
[12] F. D. Chattaway, G. Hoyle, *J. Chem. Soc., Trans.* **1923**, *123*, 654–662.
[13] B. S. Ault, L. Andrews, *J. Chem. Phys.* **1976**, *64*, 4853–4859.
[14] M. P. Bogaard, J. Peterson, A. D. Rae, *Acta Crystallogr., Sect. B: Struct. Crystallogr. Cryst. Chem.* **1981**, *37*, 1357–1359.
[15] R. T. Boeré, A. Wallace Cordes, R. T. Oakley, R. W. Reed, *J. Chem. Soc., Chem. Commun.* **1985**, *0*, 655–656.
[16] T. Chivers, J. F. Richardson, N. R. M. Smith, *Inorg. Chem.* **1985**, *24*, 2453–2458.
[17] R. T. Boere, A. W. Cordes, S. L. Craig, R. T. Oakley, R. W. Reed, *J. Am. Chem. Soc.* **1987**, *109*, 868–874.
[18] M. Jansen, S. Strojek, *Z. Naturforsch.* **1995**, *50*, 1171–1174.
[19] R. Brückner, H. Haller, M. Ellwanger, S. Riedel, *Chem. Eur. J.* **2012**, *18*, 5741–5747.
[20] F. A. Redeker, H. Beckers, S. Riedel, *Chem. Commun.* **2017**, *53*, 12958–12961.
[21] P. Voßnacker, N. Schwarze, T. Keilhack, M. Kleoff, S. Steinhauer, Y. Schiesser, M. Paven, S. Yogendra, R. Weber, S. Riedel, *ACS Sustainable Chem. Eng.* **2022**, *10*, 9525–9531.
[22] J. C. Evans, G. Y. Lo, *J. Chem. Phys.* **1966**, *44*, 3638–3639.
[23] J. Taraba, Z. Zak, *Inorg. Chem.* **2003**, *42*, 3591–3594.
[24] P. Pröhm, N. Schwarze, C. Müller, S. Steinhauer, H. Beckers, S. M. Rupf, S. Riedel, *Chem. Commun.* **2021**, *57*, 4843–4846.
[25] K. Sonnenberg, P. Pröhm, N. Schwarze, C. Müller, H. Beckers, S. Riedel, *Angew. Chem., Int. Ed.* **2018**, *57*, 9136–9140.
[26] R. Brückner, P. Pröhm, A. Wiesner, S. Steinhauer, C. Müller, S. Riedel, *Angew. Chem., Int. Ed.* **2016**, *55*, 10904–10908.
[27] R. Brückner, H. Haller, S. Steinhauer, C. Müller, S. Riedel, *Angew. Chem.* **2015**, *127*, 15800–15804.
[28] R. Brückner, H. Haller, S. Steinhauer, C. Müller, S. Riedel, *Angew. Chem., Int. Ed.* **2015**, *54*, 15579–15583.
[29] K. O. Christe, J. P. Guertin, *Inorg. Chem.* **1965**, *4*, 905–908.
[30] K. O. Christe, J. P. Guertin, *Inorg. Chem.* **1965**, *4*, 1785–1787.
[31] K. O. Christe, W. Sawodny, J. P. Guertin, *Inorg. Chem.* **1967**, *6*, 1159–1162.
[32] J. H. Miller, L. Andrews, *Inorg. Chem.* **1979**, *18*, 988–992.
[33] K. O. Christe, J. P. Guertin, *Inorg. Chem.* **1966**, *5*, 473–476.
[34] K. O. Christe, W. Sawodny, *Z. Anorg. Allg. Chem.* **1968**, *357*, 125–133.
[35] X. Zhang, K. Seppelt, *Z. Anorg. Allg. Chem.* **1997**, *623*, 491–500.

[36] B. Scheibe, S. I. Ivlev, A. J. Karttunen, F. Kraus, *Eur. J. Inorg. Chem.* **2020**, *2020*, 1319–1324.
[37] K. O. Christe, W. W. Wilson, R. V. Chirakal, J. C. P. Sanders, G. J. Schrobilgen, *Inorg. Chem.* **1990**, *29*, 3506–3511.
[38] M. S. Abdelbassit, O. J. Curnow, M. K. Dixon, M. R. Waterland, *Chem. - Eur. J.* **2019**, *25*, 11650–11658.
[39] C. Walbaum, M. Richter, U. Sachs, I. Pantenburg, S. Riedel, A.-V. Mudring, G. Meyer, *Angew. Chem.* **2013**, *125*, 12965–12968.
[40] C. Walbaum, M. Richter, U. Sachs, I. Pantenburg, S. Riedel, A.-V. Mudring, G. Meyer, *Angew. Chem., Int. Ed.* **2014**, *53*, 5233–5233.
[41] C. Walbaum, Neue Poly (Inter) Halogenide Mit Kronenether-Stabilisierten Kationen, PhD Thesis, Universität zu Köln, **2009**.
[42] M. Wolff, A. Okrut, C. Feldmann, *Inorg. Chem.* **2011**, *50*, 11683–11694.
[43] B. Scheibe, A. J. Karttunen, U. Müller, F. Kraus, *Angew. Chem., Int. Ed.* **2020**, *59*, 18116–18119.
[44] J. C. Evans, G. Y. S. Lo, *Inorg. Chem.* **1967**, *6*, 1483–1486.
[45] A. Schnittke, H. Stegemann, H. Füllbier, J. Gabrusenoks, *J. Raman Spectrosc.* **1991**, *22*, 627–631.
[46] X. Chen, M. A. Rickard, J. W. Hull, C. Zheng, A. Leugers, P. Simoncic, *Inorg. Chem.* **2010**, *49*, 8684–8689.
[47] R. Babu, G. Bhargavi, M. V. Rajasekharan, *Eur. J. Inorg. Chem.* **2015**, *2015*, 4689–4698.
[48] K. Sonnenberg, P. Pröhm, S. Steinhauer, A. Wiesner, C. Müller, S. Riedel, *Z. Anorg. Allg. Chem.* **2017**, *643*, 101–105.
[49] G. Bellucci, R. Bianchini, C. Chiappe, R. Ambrosetti, *J. Am. Chem. Soc.* **1989**, *111*, 199–202.
[50] V. Vitske, H. Herrmann, M. Enders, E. Kaifer, H.-J. Himmel, *Chem. - Eur. J.* **2012**, *18*, 14108–14116.
[51] F. B. Alhanash, N. A. Barnes, S. M. Godfrey, R. Z. Khan, R. G. Pritchard, *Polyhedron* **2013**, *65*, 102–109.
[52] H. Haller, M. Ellwanger, A. Higelin, S. Riedel, *Angew. Chem., Int. Ed.* **2011**, *50*, 11528–11532.
[53] H. Haller, M. Ellwanger, A. Higelin, S. Riedel, *Z. Anorg. Allg. Chem.* **2012**, *638*, 553–558.
[54] T. M. Beck, H. Haller, J. Streuff, S. Riedel, *Synthesis* **2014**, *46*, 740–747.
[55] H. Haller, M. Hog, F. Scholz, H. Scherer, I. Krossing, S. Riedel, *Z. Naturforsch., B: J. Chem. Sci.* **2014**, *68*, 1103–1107.
[56] H. Haller, J. Schröder, S. Riedel, *Angew. Chem., Int. Ed.* **2013**, *52*, 4937–4940.
[57] H. Haller, J. Schröder, S. Riedel, *Angew. Chem.* **2013**, *125*, 5037–5040.
[58] R. D. Chambers, W. K. Gray, G. Sandford, J. F. S. Vaughan, *J. Fluorine Chem.* **1999**, *94*, 213–215.
[59] K. O. Strømme, *Acta Chem. Scand.* **1959**, *13*, 2089–2100.
[60] R. Siepmann, H. G. von Schnering, *Z. Anorg. Allg. Chem.* **1968**, *357*, 289–298.
[61] G. B. M. Vaughan, A. J. Mora, A. N. Fitch, P. N. Gates, A. S. Muir, *J. Chem. Soc., Dalton Trans.* **1999**, 79–84.
[62] M. C. Aragoni, M. Arca, F. A. Devillanova, M. B. Hursthouse, S. L. Huth, F. Isaia, V. Lippolis, A. Mancini, H. Ogilvie, *Inorg. Chem. Commun.* **2005**, *8*, 79–82.
[63] K. Sonnenberg, P. Pröhm, C. Müller, H. Beckers, S. Steinhauer, D. Lentz, S. Riedel, *Chem. - Eur. J.* **2018**, *24*, 1072–1075.
[64] K. N. Robertson, P. K. Bakshi, T. S. Cameron, O. Knop, *Z. Anorg. Allg. Chem.* **1997**, *623*, 104–114.

[65] K. M. Fromm, R. D. Bergougnant, A. Y. Robin, *Z. Anorg. Allg. Chem.* **2006**, *632*, 828–836.
[66] C. W. Cunningham, G. R. Burns, V. McKee, *Inorg. Chim. Acta* **1990**, *167*, 135–137.
[67] M. A. Bondarenko, A. S. Novikov, V. P. Fedin, M. N. Sokolov, S. A. Adonin, *J. Coord. Chem.* **2020**, *73*, 3038–3043.
[68] M. Wolff, J. Meyer, C. Feldmann, *Angew. Chem., Int. Ed.* **2011**, *50*, 4970–4973.
[69] M. E. Easton, A. J. Ward, T. Hudson, P. Turner, A. F. Masters, T. Maschmeyer, *Chem. Eur. J.* **2015**, *21*, 2961–2965.
[70] T. Surles, L. A. Quarterman, H. H. Hyman, *J. Inorg. Nucl. Chem.* **1973**, *35*, 668–670.
[71] R. Minkwitz, R. Bröchler, R. Ludwig, *Inorg. Chem.* **1997**, *36*, 4280–4283.
[72] S. Siegel, *Acta Crystallogr.* **1956**, *9*, 493–495.
[73] W. G. Sly, R. E. Marsh, *Acta Crystallogr.* **1957**, *10*, 378–379.
[74] S. Siegel, *Acta Crystallogr.* **1957**, *10*, 380–380.
[75] K. O. Christe, C. J. Schack, *Inorg. Chem.* **1970**, *9*, 1852–1858.
[76] A. J. Edwards, G. R. Jones, *J. Chem. Soc. A* **1969**, 1936–1938.
[77] A. I. Popov, Yu. M. Kiselev, V. F. Sukhoverkhov, N. A. Chumaevsky, O. A. Krasnyanskaya, A. T. Sadikova, *Russ. J. Inorg. Chem.* **1987**, *32*, 619–622.
[78] S. I. Ivlev, R. V. Ostvald, F. Kraus, *Monatsh. Chem.* **2016**, *147*, 1661–1668.
[79] S. Ivlev, A. Karttunen, R. Ostvald, F. Kraus, *Z. Anorg. Allg. Chem.* **2015**, *641*, 2593–2598.
[80] S. Ivlev, P. Woidy, V. Sobolev, I. Gerin, R. Ostvald, F. Kraus, *Z. Anorg. Allg. Chem.* **2013**, *639*, 2846–2850.
[81] K. O. Christe, W. W. Wilson, *Inorg. Chem.* **1989**, *28*, 3275–3277.
[82] A. R. Mahjoub, A. Hoser, J. Fuchs, K. Seppelt, *Angew. Chem., Int. Ed.* **1989**, *28*, 1526–1527.
[83] A. R. Mahjoub, X. Zhang, K. Seppelt, *Chem. - Eur. J.* **1995**, *1*, 261–265.
[84] M. Möbs, T. Graubner, K. Eklund, A. J. Karttunen, F. Kraus, *Chem. - Eur. J.* **2022**, *28*, e202202466.
[85] R. Bougon, P. Charpin, J. Soriano, *C. R. Acad. Sc. Paris, Ser. C* **1971**, *272*, 565–568.
[86] L. Meazza, J. Martí-Rujas, G. Terraneo, C. Castiglioni, A. Milani, T. Pilati, P. Metrangolo, G. Resnati, *CrystEngComm* **2011**, *13*, 4427.
[87] E. M. Nour, L. H. Chen, J. Laane, *J. Phys. Chem.* **1986**, *90*, 2841–2846.
[88] G. L. Breneman, R. D. Willett, *Acta Crystallogr.* **1967**, *23*, 334–334.
[89] F. Zobi, O. Blacque, G. Steyl, B. Spingler, R. Alberto, *Inorg. Chem.* **2009**, *48*, 4963–4970.
[90] W. Gabes, *Cryst. Struct. Comm.* **1974**, *3*, 753–755.
[91] C. R. Groom, I. J. Bruno, M. P. Lightfoot, S. C. Ward, *Acta Crystallogr., Sect. B: Struct. Sci., Cryst. Eng. Mater.* **2016**, *72*, 171–179.
[92] M. Ghassemzadeh, B. Neumüller, K. Dehnicke, **2010**, DOI 10.5517/CCVJ6BW.
[93] V. F. Sukhoverkhov, N. D. Takanova, A. A. Uskova, *Zh. Neorg. Khim.* **1976**, *21*, 2245–2249.
[94] S. I. Ivlev, A. J. Karttunen, R. V. Ostvald, F. Kraus, *Chem. Commun.* **2016**, *52*, 12040–12043.
[95] J. Bandemehr, S. I. Ivlev, A. J. Karttunen, F. Kraus, *Eur. J. Inorg. Chem.* **2020**, *48*, 4568–4576.
[96] B. Schmidt, K. Sonnenberg, H. Beckers, S. Steinhauer, S. Riedel, *Angew. Chem., Int. Ed.* **2018**, *57*, 9141–9145.
[97] J. Pelletier, J. B. Caventou, *Ann. Chim. Phys.* **1819**, 142–177.
[98] P. H. Svensson, L. Kloo, *Chem. Rev.* **2003**, *103*, 1649–1684.
[99] D. Naumann, A. Meurer, *J. Fluorine Chem.* **1995**, *70*, 83–84.

[100] K. O. Christe, W. W. Wilson, G. W. Drake, M. A.Petrie, J. A. Boatz, *J. Fluorine Chem.* **1998**, *88*, 185–189.
[101] K. O. Christe, D. Naumann, *Inorg. Chem.* **1973**, *12*, 59–62.
[102] J. Shamir, I. Yaroslavsky, *Isr. J. Chem.* **1969**, *7*, 495–497.
[103] A. R. Mahjoub, K. Seppelt, *Angew. Chem., Int. Ed.* **1991**, *30*, 323–324.
[104] K. Seppelt, *Acc. Chem. Res.* **2003**, *36*, 147–153.
[105] K. O. Christe, J. P. Guertin, W. Sawodny, *Inorg. Chem.* **1968**, *7*, 626–628.
[106] C. J. Adams, *Inorg. Nucl. Chem. Lett.* **1974**, *10*, 831–835.
[107] K. O. Christe, J. C. P. Sanders, G. J. Schrobilgen, W. W. Wilson, *J. Chem. Soc., Chem. Commun.* **1991**, *0*, 837–840.
[108] A.-R. Mahjoub, K. Seppelt, *Angew. Chem., Int. Ed.* **1991**, *30*, 876–878.
[109] K. O. Christe, W. W. Wilson, G. W. Drake, D. A. Dixon, J. A. Boatz, R. Z. Gnann, *J. Am. Chem. Soc.* **1998**, *120*, 4711–4716.
[110] Y.-Q. Wang, Z.-M. Wang, C.-S. Liao, C.-H. Yan, *Acta Crystallogr., Sect. C: Cryst. Struct. Commun.* **1999**, *55*, 1503–1506.
[111] G. J. Visser, A. Vos, *Acta Crystallogr.* **1964**, *17*, 1336–1337.
[112] R. C. L. Mooney, *Z. Kristallogr. - Cryst. Mater.* **1939**, *100*, 519–529.
[113] R. W. G. Wyckoff, *J. Am. Chem. Soc.* **1920**, *42*, 1100–1116.
[114] C. Rømming, J. Goa, H. Colldahl, L. G. Sillén, B. Zaar, E. Diczfalusy, *Acta Chem. Scand.* **1958**, *12*, 668–677.
[115] H. Kobayashi, R. Kato, A. Kobayashi, G. Saito, M. Tokumoto, H. Anzai, T. Ishiguro, *Chem. Lett.* **1986**, *15*, 89–92.
[116] G. Shilov, O. Kazheva, O. D'yachenko, M. Chernov'yants, S. Simonyan, V. Gol'eva, A. Pyshchev, *Russ. J. Phys. Chem.* **2002**, *76*, 1295–1301.
[117] A. S. Romanov, M. Bochmann, *Organometallics* **2015**, *34*, 2439–2454.
[118] J. Martí-Rujas, L. Meazza, G. K. Lim, G. Terraneo, T. Pilati, K. D. M. Harris, P. Metrangolo, G. Resnati, *Angew. Chem., Int. Ed.* **2013**, *52*, 13444–13448.
[119] T. Ejima, J. L. de Boer, A. Vos, *Acta Crystallogr.* **1963**, *16*, 243–247.
[120] B. Burgenmeister, K. Sonnenberg, S. Riedel, I. Krossing, *Chem. - Eur. J.* **2017**, *23*, 11312–11322.
[121] D. Hausmann, A. Eich, C. Feldmann, *J. Mol. Struct.* **2018**, *1166*, 159–163.
[122] C. L. R. Mooney, *Z. Kristallogr. - Cryst. Mater.* **1938**, *98*, 324–333.
[123] T. J. Emge, H. H. Wang, P. C. W. Leung, P. R. Rust, J. D. Cook, P. L. Jackson, K. Douglas. Carlson, J. M. Williams, M. Hwan. Whangbo, *J. Am. Chem. Soc.* **1986**, *108*, 695–702.
[124] P. C. W. Leung, T. J. Emge, A. J. Schultz, M. A. Beno, K. D. Carlson, H. H. Wang, M. A. Firestone, J. M. Williams, *Solid State Commun.* **1986**, *57*, 93–97.
[125] E. Laukhina, J. Vidal-Gancedo, S. Khasanov, V. Tkacheva, L. Zorina, R. Shibaeva, J. Singleton, R. Wojciechowski, J. Ulanski, V. Laukhin, J. Veciana, C. Rovira, *Adv. Mater.* **2000**, *12*, 1205–1210.
[126] E. Laukhina, J. Vidal-Gancedo, V. Laukhin, J. Veciana, I. Chuev, V. Tkacheva, K. Wurst, C. Rovira, *J. Am. Chem. Soc.* **2003**, *125*, 3948–3953.
[127] M. Watanabe, Y. Noda, H. Taniguchi, *J. Low Temp. Phys.* **2006**, *142*, 163–166.
[128] Y. Yagi, A. I. Popov, *J. Inorg. Nucl. Chem.* **1967**, *29*, 2223–2230.
[129] M. S. Abdelbassit, O. J. Curnow, *Chem. - Eur. J.* **2019**, *25*, 13294–13298.
[130] H. Endres, M. Hiller, H. J. Keller, *Z. Naturforsch. B* **1985**, *40*, 1664–1671.
[131] A. Parlow, H. Hartl, *Z. Naturforsch. B* **1985**, *40*, 45–52.
[132] S. Soled, G. B. Carpenter, *Acta Crystallogr., Sect. B: Struct. Crystallogr. Cryst. Chem.* **1973**, *29*, 2556–2559.
[133] J. E. Davies, E. K. Nunn, *J. Chem. Soc. D* **1969**, 1374a.

[134] D. Hausmann, R. Köppe, S. Wolf, P. W. Roesky, C. Feldmann, *Dalton Trans.* **2016**, *45*, 16526–16532.
[135] T. J. Emge, H. H. Wang, M. A. Beno, P. C. W. Leung, M. A. Firestone, H. C. Jenkins, J. D. Cook, J. M. Williams, E. L. Venturini, *Inorg. Chem.* **1985**, *24*, 1736–1738.
[136] H. Kobayashi, R. Kato, A. Kobayashi, G. Saito, M. Tokumoto, H. Anzai, T. Ishiguro, *Chem. Lett.* **1985**, *14*, 1293–1296.
[137] O. N. Kazheva, G. G. Aleksandrov, O. A. D'yachenko, M. S. Chernov'yants, S. S. Simonyan, E. O. Lykova, *Russ. J. Coord. Chem.* **2003**, *29*, 819–827.
[138] I.-E. Parigoridi, G. J. Corban, S. K. Hadjikakou, N. Hadjiliadis, N. Kourkoumelis, G. Kostakis, V. Psycharis, C. P. Raptopoulou, M. Kubicki, *Dalton Trans.* **2008**, 5159.
[139] L. Mann, Investigation of Polybromides, Non-Classical Polyinterhalides Based on ICl and IBr, and Fluorinated [PPN]+-Salts, PhD thesis, Freie Universität Berlin, **2018**.
[140] M. S. Abdelbassit, O. J. Curnow, M. K. Dixon, M. R. Waterland, *Chem. - Eur. J.* **2019**, *25*, 11659–11669.
[141] K. O. Christe, *Inorg. Chem.* **1972**, *11*, 1215–1219.
[142] J. Cornog, E. E. Bauer, *J. Am. Chem. Soc.* **1942**, *64*, 2620–2624.
[143] Y. Yagi, A. I. Popov, *Inorg. Nucl. Chem. Lett.* **1965**, *1*, 21–24.
[144] A. Parlow, H. Hartl, *Acta Crystallogr. B, Struct. Crystallogr. Cryst. Chem.* **1979**, *35*, 1930–1933.
[145] L. Mann, *Master Thesis*, Albert-Ludwigs Universität, Freiburg, **2013**.
[146] K. Sonnenberg, L. Mann, F. A. Redeker, B. Schmidt, S. Riedel, *Angew. Chem., Int. Ed.* **2020**, *59*, 5464–5493.
[147] L. Mann, P. Voßnacker, C. Müller, S. Riedel, *Chem. Eur. J.* **2017**, *23*, 244–249.
[148] R. Minkwitz, M. Berkei, R. Ludwig, *Inorg. Chem.* **2001**, *40*, 25–28.
[149] M. C. Aragoni, M. Arca, F. A. Devillanova, M. B. Hursthouse, S. L. Huth, F. Isaia, V. Lippolis, A. Mancini, G. Verani, *Eur. J. Inorg. Chem.* **2008**, *2008*, 3921–3928.
[150] G. A. Olah, M. B. Comisarow, *J. Am. Chem. Soc.* **1968**, *90*, 5033–5034.
[151] S. K. Bramble, P. A. Hamilton, *J. Chem. Soc., Faraday Trans.* **1990**, *86*, 2009–2013.
[152] F. Cacace, G. de Petris, F. Pepi, M. Rosi, A. Sgamellotti, *Rapid Commun. Mass Spectrom.* **1998**, *12*, 1911–1913.
[153] R. Minkwitz, J. Nowicki, H. Härtner, W. Sawodny, *Spectrochim. Acta, Part A* **1991**, *47*, 1673–1676.
[154] S. Seidel, K. Seppelt, *Angew. Chem., Int. Ed.* **2000**, *39*, 3923–3925.
[155] K. O. Christe, W. Sawodny, *Inorg. Chem.* **1967**, *6*, 313–318.
[156] R. J. Gillespie, M. J. Morton, *Inorg. Chem.* **1970**, *9*, 616–618.
[157] H. Lynton, J. Passmore, *Can. J. Chem.* **1971**, *49*, 2539–2543.
[158] B. Scheibe, R. Haiges, S. I. Ivlev, A. J. Karttunen, U. Müller, K. O. Christe, F. Kraus, *Eur. J. Inorg. Chem.* **2020**, *2020*, 4483–4496.
[159] G. Frenking, W. Koch, *Inorg. Chem.* **1990**, *29*, 4513–4517.
[160] K. O. Christe, R. D. Wilson, *Inorg. Nucl. Chem. Lett.* **1973**, *9*, 845–847.
[161] K. O. Christe, X. Zhang, J. A. Sheehy, R. Bau, *J. Am. Chem. Soc.* **2001**, *123*, 6338–6348.
[162] W. W. Wilson, K. O. Christe, *J. Fluorine Chem.* **1982**, *21*, 7.
[163] K. O. Christe, *Inorg. Chem.* **1973**, *12*, 1580–1587.
[164] K. O. Christe, W. W. Wilson, E. C. Curtis, *Inorg. Chem.* **1983**, *22*, 3056–3060.
[165] R. J. Gillespie, M. J. Morton, *Inorg. Chem.* **1972**, *11*, 586–591.
[166] W. W. Wilson, R. C. Thompson, F. Aubke, *Inorg. Chem.* **1980**, *19*, 1489–1493.
[167] K. O. Christe, R. Bau, D. Zhao, *Z. Anorg. Allg. Chem.* **1991**, *593*, 46–60.
[168] K. O. Christe, D. A. Dixon, R. Minkwitz, *Z. Anorg. Allg. Chem.* **1992**, *612*, 51–55.
[169] K. O. Christe, C. J. Schack, *Inorg. Chem.* **1970**, *9*, 2296–2299.
[170] H. A. Carter, F. Aubke, *Can. J. Chem.* **1970**, *48*, 3456–3459.

[171] A. Vij, F. S. Tham, V. Vij, W. W. Wilson, K. O. Christe, *Inorg. Chem.* **2002**, *41*, 6397–6403.
[172] M. D. Lind, K. O. Christe, *Inorg. Chem.* **1972**, *11*, 608–612.
[173] R. J. Gillespie, G. J. Schrobilgen, *Inorg. Chem.* **1974**, *13*, 1230–1235.
[174] S. I. Ivlev, A. J. Karttunen, M. R. Buchner, M. Conrad, F. Kraus, *Angew. Chem., Int. Ed.* **2018**, *57*, 14640–14644.
[175] R. J. Gillespie, M. J. Morton, *J. Mol. Spectrosc.* **1969**, *30*, 178–183.
[176] R. J. Gillespie, J. B. Milne, *Inorg. Chem.* **1966**, *5*, 1577–1582.
[177] C. Chung, G. H. Cady, *Inorg. Chem.* **1972**, *11*, 2528–2531.
[178] R. Faggiani, R. J. Gillespie, R. Kapoor, C. J. L. Lock, J. E. Vekris, *Inorg. Chem.* **1988**, *27*, 4350–4355.
[179] M. Müller, M. Albrecht, V. Gossen, T. Peters, A. Hoffmann, G. Raabe, A. Valkonen, K. Rissanen, *Chem. Eur. J.* **2010**, *16*, 12446–12453.
[180] J. Passmore, P. Taylor, T. Whidden, P. S. White, *Can. J. Chem.* **1979**, *57*, 968–973.
[181] S. Hoyer, Strukturchemie von Iodverbindungen in den Oxidationsstufen +1/7 bis +5, Dissertation, Freie Universität Berlin, **2004**.
[182] Thomas. Birchall, R. D. Myers, *Inorg. Chem.* **1981**, *20*, 2207–2211.
[183] J. Shamir, R. Rafaeloff, *Spectrochim. Acta, Part A* **1973**, *29*, 873–878.
[184] J. Shamir, M. Lustig, *Inorg. Chem.* **1973**, *12*, 1108–1112.
[185] T. Birchall, R. D. Myers, *Inorg. Chem.* **1983**, *22*, 1751–1756.
[186] Thomas. Birchall, R. D. Myers, *Inorg. Chem.* **1982**, *21*, 213–217.
[187] W. W. Wilson, F. Aubke, *Inorg. Chem.* **1974**, *13*, 326–332.
[188] K. O. Christe, W. Sawodny, *Inorg. Chem.* **1967**, *6*, 1783–1788.
[189] W. W. Wilson, K. O. Christe, *Inorg. Chem.* **1989**, *28*, 4172–4175.
[190] K. O. Christe, W. W. Wilson, R. D. Wilson, R. Bau, J. A. Feng, *J. Am. Chem. Soc.* **1990**, *112*, 7619–7625.
[191] *APEX3 V2019.11-2*, Bruker AXS Inc., Madison, Wisconsin, USA, **2019**.
[192] G. M. Sheldrick, *Acta Crystallogr.* **2015**, *A71*, 3–8.
[193] G. M. Sheldrick, *Acta Crystallogr.* **2015**, *C71*, 3–8.
[194] C. B. Hübschle, G. M. Sheldrick, B. Dittrich, *J. Appl. Crystallogr.* **2011**, *44*, 1281–1284.
[195] S. I. Ivlev, M. Conrad, F. Kraus, *Z. Kristallogr. - Cryst. Mater.* **2019**, *234*, 415–418.
[196] K. Brandenburg, H. Putz, *Diamond - Crystal and Molecular Structure Visualization, V 4.6.8*, Crystal Impact GbR, Bonn, **2022**.
[197] G. Beata, G. Perego, B. Civalleri, *J. Comput. Chem.* **2019**, *40*, 2329–2338.
[198] “CRYSPLOT - A modern and easy to use visualization environment for plotting properties of crystalline solids as computed by means of the CRYSTAL code.,” can be found under http://crysplot.crystalsolutions.eu/index.html, **2022**.
[199] G. Kabisch, M. Klose, *J. Raman Spectrosc.* **1978**, *7*, 311–315.
[200] R. Ahlrichs, M. Bär, M. Häser, H. Horn, C. Kölmel, *Chem. Phys. Lett.* **1989**, *162*, 165–169.
[201] *TURBOMOLE V7.7, a Development of University of Karlsruhe and Forschungszentrum Karlsruhe GmbH, 1989-2007, TURBOMOLE GmbH, since 2007.*, **2022**.
[202] C. Adamo, V. Barone, *J. Chem. Phys.* **1999**, *110*, 6158–6170.
[203] J. P. Perdew, K. Burke, M. Ernzerhof, *Phys. Rev. Lett.* **1996**, *77*, 3865–3868.
[204] F. Weigend, R. Ahlrichs, *Phys. Chem. Chem. Phys.* **2005**, *7*, 3297–3305.
[205] K. A. Peterson, D. Figgen, E. Goll, H. Stoll, M. Dolg, *J. Chem. Phys.* **2003**, *119*, 11113–11123.
[206] F. Weigend, *Phys. Chem. Chem. Phys.* **2002**, *4*, 4285–4291.
[207] K. Eichkorn, O. Treutler, H. Öhm, M. Häser, R. Ahlrichs, *Chem. Phys. Lett.* **1995**, *240*, 283–290.
[208] M. Sierka, A. Hogekamp, R. Ahlrichs, *J. Chem. Phys.* **2003**, *118*, 9136–9148.

[209] A. Klamt, G. Schürmann, *J. Chem. Soc., Perkin Trans. 2* **1993**, 799–805.
[210] G. Knizia, *J. Chem. Theory Comput.* **2013**, *9*, 4834–4843.
[211] A. Erba, J. K. Desmarais, S. Casassa, B. Civalleri, L. Donà, I. J. Bush, B. Searle, L. Maschio, L. Edith-Daga, A. Cossard, C. Ribaldone, E. Ascrizzi, N. L. Marana, J.-P. Flament, B. Kirtman, *J. Chem. Theory Comput.* **2023**, *19*, 6891–6932.
[212] S. I. Ivlev, K. Gaul, M. Chen, A. J. Karttunen, R. Berger, F. Kraus, *Chem. - Eur. J.* **2019**, *25*, 5793–5802.
[213] A. J. Karttunen, T. Tynell, M. Karppinen, *J. Phys. Chem. C* **2015**, *119*, 13105–13114.
[214] S. S. Rudel, T. Graubner, A. J. Karttunen, F. Kraus, *Z. Anorg. Allg. Chem.* **2020**, *646*, 1396–1402.
[215] S. Grimme, J. Antony, S. Ehrlich, H. Krieg, *J. Chem. Phys.* **2010**, *132*, 154104.
[216] S. Grimme, A. Hansen, J. G. Brandenburg, C. Bannwarth, *Chem. Rev.* **2016**, *116*, 5105–5154.
[217] H. J. Monkhorst, J. D. Pack, *Phys. Rev. B* **1976**, *13*, 5188–5192.
[218] L. Maschio, B. Kirtman, M. Rérat, R. Orlando, R. Dovesi, *J. Chem. Phys.* **2013**, *139*, 164102.
[219] C. M. Zicovich-Wilson, F. Pascale, C. Roetti, V. R. Saunders, R. Orlando, R. Dovesi, *J. Comput. Chem.* **2004**, *25*, 1873–1881.
[220] F. Pascale, C. M. Zicovich-Wilson, F. López Gejo, B. Civalleri, R. Orlando, R. Dovesi, *J. Comput. Chem.* **2004**, *25*, 888–897.
